# The Short-Term Psychological Impact of the COVID-19 Pandemic in Psychiatric Patients: Evidence for Differential Emotion and Symptom Trajectories in Belgium

**DOI:** 10.5334/pb.1028

**Published:** 2021-06-21

**Authors:** Egon Dejonckheere, Marlies Houben, Evelien Schat, Eva Ceulemans, Peter Kuppens

**Affiliations:** 1 KU Leuven – Faculty of Psychology and Educational Sciences, Belgium; 2 Faculty of Psychology and Educational Sciences, KU Leuven, Tiensestraat 102, Leuven, 3000, Belgium; 3 Mind-Body Research and Center for Contextual Psychiatry, Department of Neurosciences, KU Leuven, Kapucijnenvoer 7, Leuven, 3000, Belgium

**Keywords:** COVID-19, mental health crisis, resilience, experience sampling methods, psychological well-being

## Abstract

The spread of COVID-19 and the implementation of various containment strategies across the world have seriously disrupted people’s everyday life, and it is especially uncertain what the psychological impact of this pandemic will be for vulnerable individuals, such as psychiatric (ex-)patients. Governments fear that this virus outbreak may prelude a major mental health crisis, and psychiatrists launch critical calls to flatten an upcoming mental ill-health surge. Here, we aim to add nuance to the idea that we are heading towards a mental health pandemic and that psychiatric populations will unavoidably (re)develop psychopathology. Despite being subjected to the same challenges posed by COVID-19, we argue that people with a history of psychiatric illness will psychologically deal with this adversity in different ways. To showcase the short-term differential impact of COVID-19 on patients’ mental health, we present the day-to-day emotion and symptom trajectories of different psychiatric patients that took part in an experience sampling study before, during, and after the start of the first wave of the COVID-19 pandemic in March 2020 and associated lockdown measures in Belgium. Piecewise regression models show that not all patients’ psychological well-being is affected to a similar degree. As such, we argue that emphasizing human resilience, also among the more vulnerable in society, may be opportune in these unsettling times.

## Introduction

The COVID-19 pandemic, together with the ensuing isolation or lockdown measures taken by a greater number of countries, poses unprecedented challenges in various life domains (Druss, 2020). Whether it is the fear of virus contagion, the restriction of real-life contact with family and friends, or the economic insecurity associated with unemployment: Drastic changes in physical, social and professional life are thought to have major direct or indirect repercussions for our psychological health as expressed by public surveys and expert panels (Holmes et al., 2020). Consequently, both researchers and policy makers call for a close and timely monitoring of people’s mental well-being (United Nations, 2020), as they anticipate a steep increase in psychological problems, reflected in, for example, elevated suicide levels and self-harm prevalence (based on empirically established suicide models [Kawohl & Nordt, 2020] and preliminary case evidence [Sahoo et al., 2020]), increased alcohol abuse and relapse (personal opinion based on scientific literature review; Clay & Parker, 2020), or domestic violence (extrapolations based on previous natural disasters’ effects on family violence; Campbell, 2020). Moreover, in several countries, national assessments already reported preliminary evidence for a significant surge in depression and anxiety-related complaints in the general population (Ambraw et al., unpublished data; Chaix et al., 2020; Qiu et al., 2020), underscoring the potential psychological impact of the coronavirus outbreak and related quarantine measures.

Despite the pandemic’s global proportions, it is feared that this outbreak may disproportionately affect vulnerable and at-risk individuals (Druss, 2020), and people with a pre-existing psychiatric illness are thought to be among the worst affected in the community (Kavoor, 2020). Based on a preliminary literature review about the potential psychological impact of COVID-19, Rajkumar (2020) expects that, while this pandemic may trigger temporary uncertainty, stress and worry in healthy individuals, for patients who suffer from mental disorders, the threat of this virus is thought to add burden to their psychiatric condition. As a consequence, this pandemic could cultivate the further deterioration of their mental health status. Similarly, for ex-patients or recovered individuals, initial case studies illustrate that this pandemic may prelude a new episode of debilitating psychiatric symptoms or instigate a relapse into old, yet maladaptive coping strategies (Columb, Hussain, & O’Gara, 2020; Mehra et al., 2020). Compounded by a limited or interrupted access to mental health facilities as a result of physical distancing measures (Duan & Zhu, 2020; Kavoor, Chakravarthy, & John, 2020), an executive summary of the United Nations (2020) warns that *the coronavirus pandemic could have the seeds of a major mental health crisis*, in which many at-risk individuals (re-)experience psychological complaints. In response, researchers and health care professionals launch critical calls to urgently *flatten the mental-ill health curve* (Carbone, 2020; Xiang et al., 2020).

Notwithstanding long-term monitoring of people’s psychological well-being will be imperative in the aftermath of this virus outbreak, we argue that emphasizing human resilience (Bonanno & Burton, 2013; Kalisch et al., 2017), also among the more vulnerable in society, may be opportune in these unsettling times. The premonition of a mental health *pandemic* or *crisis* may falsely create the impression that this unusual event will psychologically affect all people with (a history of) mental illness in a similar devastating way. However, decades of research decisively demonstrate critical person-situation interactions in psychological adjustment after exposure to an impactful stressor, a pattern that has been established in both healthy and at-risk populations, and for different types of hardship (Bonanno & Burton, 2013; Fergusson et al., 2014; Waugh & Koster, 2015). Although COVID-19 shows new and unique stressor features (e.g., the invisibility of the virus, its global proportions; Polizzi, Lynn & Perry, 2020), this pandemic also shows many characteristics shared with other commonly investigated stressors (Anisman & Merali, 1999), such as chronicity (e.g., war or famine; Lothe & Heggen, 2003; McAndrew et al. 2017), uncontrollability and unpredictability (e.g., tsunamis, hurricanes or earthquakes; Norris, Tracy & Galea, 2009; Tsuboya et al., 2016; Sasaki et al., 2019). Therefore, based on this body of research, we expect that, despite being subjected to the same challenges posed by the COVID-19 pandemic, at-risk individuals will psychologically deal with this adversity in different ways, depending on their own personal situation and their available coping abilities (e.g., Bonanno, Westphal, & Mancini, 2011). As such, we hypothesize that at least a significant subgroup of people with existing psychiatric difficulties will resiliently cope with this unusual stressor, showing either no or limited psychological distress in response to the pandemic, or temporary psychological distress that constitutes a normal reaction to an abnormal event. In contrast, a second subgroup may develop sustained stress-related mental dysfunction, with (sub)clinical symptoms aggravating over time.

## Participants

To showcase the short-term differential impact of COVID-19 on at-risk people’s mental health, we analyzed the day-to-day mood and symptom trajectories of four single case participants (4 women; *M*_age_ = 39, *SD*_age_ = 17.17) who took part in a longitudinal and tailored experience sampling (ESM) study (for a general introduction on ESM, see Myin-Germeys et al., 2018). Participants all had a history of psychiatric illness and were contacted in February / March 2019 during the rehabilitation phase of their psychiatric hospitalization in a large Belgian clinic. They were part of a subject pool for another clinical ESM study (of which the study details are described elsewhere; Heininga et al., 2019), and we invited them without obligation to take part in a longitudinal and idiosyncratic adaptation of this protocol (more information below).

In total, 12 participants began the tailored ESM protocol. However, five participants had already discontinued the study before the first Belgian lockdown ensued (i.e., March, 13^th^) for reasons unrelated to COVID-19 (e.g., because the protocol was incompatible with their jobs). Another three participants had no ESM data directly following the start of the first lockdown due to temporary drop-out (e.g., due to temporary loss of the study phone) or technical issues. Consequently, only four participants were able to provide adequate time series data around the time the first lockdown was issued.

During an intake session prior to the actual ESM protocol, participants’ clinical status was evaluated using the Dutch version of the Structured Clinical Interview for DSM-IV Axis I disorders (SCID-I; Van Groenestijn et al., 1999) and DSM-IV Axis II disorders (SCID-II; Weertman, Arntz, & Kerkhofs, 2000). Interrater reliability, based on a comparison of the two first-authors’ ratings of seven randomly selected audiotaped interviews, was excellent, both at the level of diagnosis (κ = .93) and individual symptoms (κ = .92). For Patient 1 intake diagnoses comprised bipolar disorder (Type II) and panic disorder with agoraphobia. Patient 2 suffered from major depressive disorder and panic disorder with agoraphobia at the time of intake. Patient 3 was diagnosed with major depressive disorder, remitted substance abuse (amphetamines), panic disorder with agoraphobia, and borderline personality disorder. Finally, for Patient 4, we could not conclude any formal SCID-I or SCID-II diagnoses, but her symptoms were all related to burn-out, together with somatization-related complaints (e.g., extreme fatigue and unexplained pain symptoms). During the ESM protocol, no information about patients’ mental health status was collected in the form of clinical diagnoses. For a detailed summary of all participant characteristics, we refer to ***Table 1***.

**Table 1:** Summary of all participant characteristics.


INFORMATION	PATIENT 1	PATIENT 2	PATIENT 3	PATIENT 4

Baseline information				

Gender	Female	Female	Female	Female

Age	58	20	30	48

Intake diagnoses	Bipolar disorder (Type II)	Major depressive disorder	Major depressive disorder	Burn-out

	Panic disorder with agoraphobia	Panic disorder with agoraphobia	Panic disorder with agoraphobia	Somatization-related complaints

			Substance abuse (remitted)	

			Borderline personality disorder	

ESM information				

Days of ESM since intake	464	406	393	393

Number of items	14	16	16	12

Waking hours	9AM–8PM	8AM–9PM	8.30AM–9PM	8AM–8PM

Compliance	60.09%	40.00%	82.68%	46.71%


## Procedure and Materials

All four participants took part in a tailored and longitudinal ESM protocol that aimed to detect personalized early warning signals for a gradual or abrupt transition in patients’ mental well-being (e.g., Wichers et al., 2016). Because we started recruiting participants in February 2019, we were able to capture changes in their well-being in response to the unfolding of this pandemic at the beginning of 2020. Contrary to other corona-related ESM studies (e.g., Fried, Papanikolaou, & Epskamp, 2020), the available information about their mental health prior to the COVID-19 outbreak provides critical baseline data and enables unique insights into the dynamical nature of idiosyncratic symptomatology directly in response to the Belgian COVID-19 spread (first wave in March 2020) and the associated lockdown measures.

As part of the ESM study, participants carried a study smartphone with our custom-made software for ESM studies (www.m-path.io; www.mobileq.org; Meers et al., 2019) in their everyday lives, and we instructed them to report on their momentary level of various idiographic symptoms and emotions, four times per day. The specific content of momentary surveys was determined in agreement with each patient via clinical interviews with the first two authors of this paper, ensuring optimal personal relevance and excellent face validity of the survey items for the participant in question. The full quantitative item list for each participant can be found in Supplemental Materials 1, together with the exact phrasing of all questions. All quantitative items were rated on a continuous slider ranging from 0 (*not at all*) to 100 (*very much*). Categorical (e.g., *Who are you with right now?*) and binary items (e.g., *Did you take your medication last night?*) are not presented, because these time series data are hard to visualize.

Adopting a time-contingent semi-random sampling protocol, each patient’s waking hours (excluding moments where therapy sessions were scheduled) were divided into four time blocks, in which a questionnaire was randomly triggered. This typically resulted in one survey being scheduled in the morning before 9AM, one around noon, and two surveys after 4.30PM. This approach allowed us to comprehensively sample an entire day in people’s lives (ensuring ecological representativeness of the data), without the surveys being too predictable (which could compromise natural behavior and introduce interference of the protocol; Dejonckheere & Erbas, 2021). Within a day, the average time interval between two consecutive surveys was 3 hours and 14 minutes per participant (*SD* = 34 minutes), and participants’ compliance at the survey-level ranged from 40.0% to 82.7% for the time period under study (*M* = 57.4%, *SD* = 18.8%). Participants received €20 per week of ESM if they completed 75% of the momentary surveys or more (with a €2 deduction per 5% completed less).

## Statistical Analyses

The data we analyzed, span from January to April 2020, and thus provide information regarding their mood and symptom levels several weeks before and after the first major lockdown was proclaimed in Belgium (March, 13^th^). To detect meaningful changes in patients’ symptom and emotion levels in response to the first COVID-19 lockdown, we fitted multiple piecewise regression models for all participants’ tracked features. This approach enabled us to statistically evaluate critical changes in temporal mood and symptom trajectories before and after the first COVID-19 lockdown. To eliminate situational within-day fluctuations, we averaged all symptom or emotion rating scores per day. The generic model structure in which we predict the daily severity of a particular *symptom* (e.g., feeling depressed) at day *d* is presented below:

\begin{array}{l}
sympto{m_d}= {\beta _0} + {\beta _e}\;event + \;{\beta _d}\;da{y_d} + \;{\beta _{e*d}}\;event*da{y_d}\\
\qquad\qquad\qquad\ + \;{\beta _l}\;sympto{m_{d - 1}}
\end{array}

For participant 1 to 3, the effect β_e_ of dummy *event* denotes the change in symptom severity on the day the lockdown was issued. For participant 4, *event* refers to a self-reported intense family conflict (see below for more information). Next, β_d_ represents the *day* trend of a particular symptom before the event occurred. We centered *day* around that *event* for an intuitive interpretation of all coefficients, meaning that the event day was day zero. Critical to our research question, the effect β_e*d_ demonstrates how this *day* trend shifts before and after the *event*, and denotes change in the trajectory of the examined features due to the announced lockdown (for patients 1 to 3) or family conflict (for patient 4). Finally, to take into account the serial dependency in participants’ symptom or emotion time series, we always added a lagged symptom version *symptom_d-1_* and evaluated its effect β_l_. In this way, we could establish event * day interactions above and beyond the self-predictiveness of patients’ symptoms or emotions. Given that we tested a considerable number of models per patient, we controlled for multiple testing per participant following the False Discovery Rate (FDR) procedure of Benjamini and Hochberg (1995).

## Results

**Figure 1:**
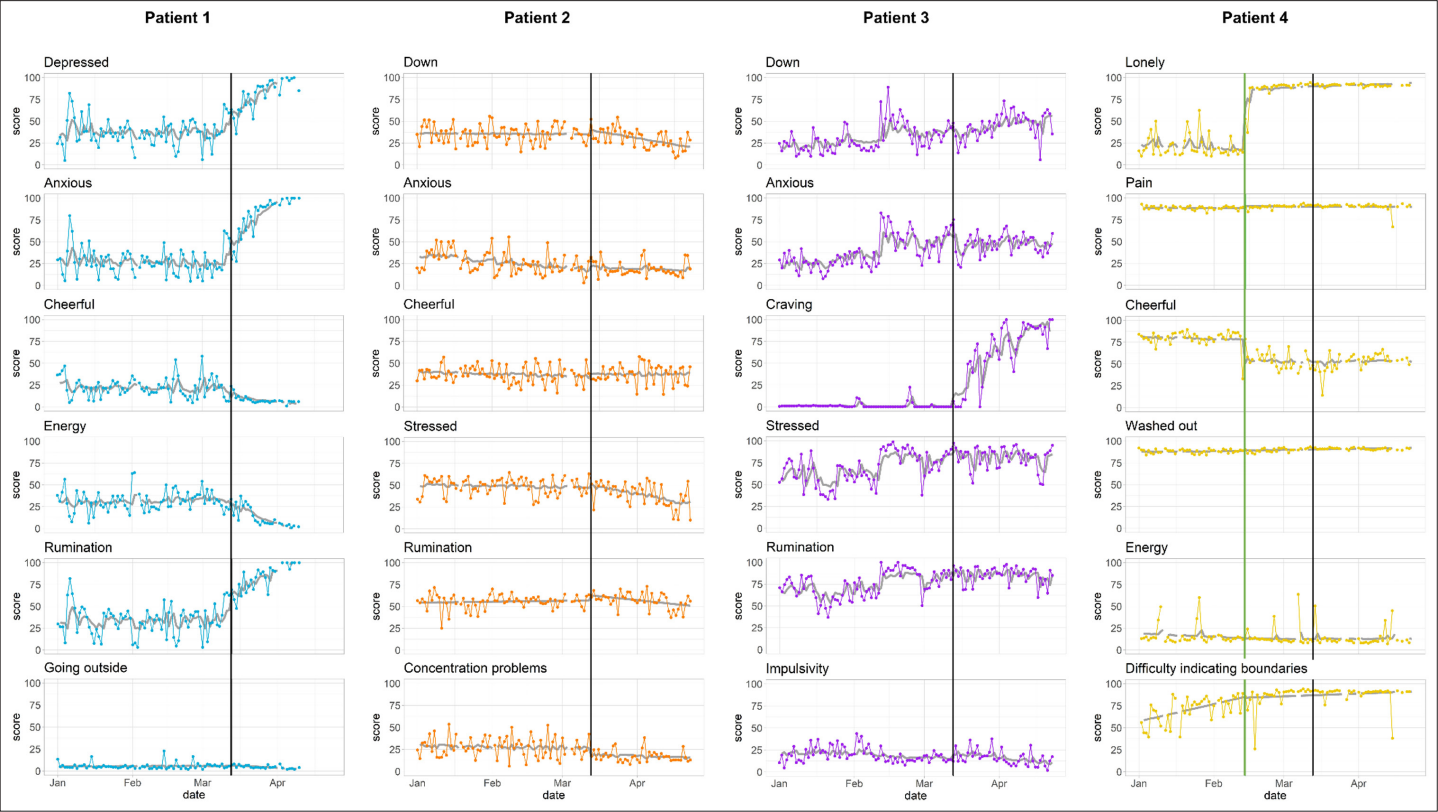
**Different mood and symptom trajectories in response to the Belgian COVID-19 lockdown measures**. Panel A depicts six case-representative symptoms or emotions for each subject. All momentary states are rated on continuous slider scales ranging from 0 (*not at all*) to 100 (*very much*). In all graphs, the colored line represents the original time series data (averaged per day to get an indication of daily emotion and symptom fluctuations); the grey line shows the model-based predicted time-series, allowing to assess model fit. The black vertical line denotes the beginning of the lockdown period (issued on March 13^th^). For Patient 4, the green line depicts a self-reported family conflict (February 14^th^).

For each subject, the temporal trajectories of six case-representative emotions or symptoms are visualized in ***Figure 1*** (see Supplemental Materials 2 for a graphical overview of all assessed features and ***Table 2*** for a numerical summary of all piecewise regression models).

**Table 2:** Summary of all piecewise regression models.


PATIENT 1					PATIENT 2					PATIENT 3					PATIENT 4				
			
Items	β_e_	β_d_	β_e*d_	β_l_	Items	β_e_	β_d_	β_e*d_	β_l_	Items	β_e_	β_d_	β_e*d_	β_l_	Items	β_e_	β_d_	β_e*d_	β_l_

**Depressive features**																			

Depressed	13.97	0.00	1.33*	0.31*	Down	4.49	–0.01	–0.44	0.03	Down	–3.62	0.20*	0.07	0.36***					

Sad	12.47	–0.09	0.19	0.29*	Guilt	3.96	–0.11	0.07	–0.04	Frustrated	9.23	0.03	0.08	0.31**	Anger	–0.49	0.12	–0.05	0.09

Cheerful	–5.32	–0.02	–0.24	0.32*	Cheerful	2.87	–0.08	0.08	–0.11	Cheerful	–2.18	–0.07	0.06	0.51***	Cheerful	–20.71**	–0.08	0.08	0.13

Anhedonia	12.98	–0.11	0.79	0.37*						Anhedonia	2.86	0.10	–0.10	0.38***					

Self-esteem	–4.93	–0.02	–0.38	0.33*	Self-esteem	–1.37	0.03	–0.14	–0.10	Self-esteem	–3.80	–0.05	0.01	0.31**					

Rumination	19.21*	0.03	0.92	0.33*	Rumination	5.76	0.04	–0.32	0.00	Rumination	0.90	0.17*	–0.24	0.49***					

Suppression	13.95	0.11	0.50	0.41**	Suppression	2.00	–0.10	0.14	–0.02	Suppression	–8.33	0.23*	0.04	0.55***					

Energy	–5.26	0.05	–0.93*	0.22	Tired	0.19	–0.04	–0.19	0.08	Energy	–1.33	–0.06	0.05	0.52***	Energy	–0.51	–0.13	0.13	0.15

Conc. problems	–4.58	–0.02	0.87**	0.56***	Conc. problems	–8.20	–0.04	–0.06	–0.14	Conc. problems	0.75	0.15*	–0.24	0.45***	Conc. problems	1.25	0.03	–0.03	0.17

Social pressure	14.14*	0.00	–0.95*	0.88***	Unease	–0.93	–0.06	0.06	0.12										

**Anxiety features**																			

Anxious	17.91*	–0.04	1.79**	0.28*	Anxious	2.62	–0.21	0.14	0.16	Anxious	–7.79	0.30**	–0.21	0.45***					

Stressed	12.19	–0.03	2.70***	–0.01	Stressed	2.09	–0.04	–0.43	0.11	Stressed	–0.66	0.19*	–0.22	0.52***					

Relaxed	–8.16	–0.02	–0.61	0.18	Relaxed	–1.11	0.05	0.01	0.05	Relaxed	–2.19	–0.13*	0.05	0.31**	Relaxed	–14.61*	–0.02	–0.12	0.11

Going outside	0.69	0.01	–0.18	–0.16	Crowded	–3.22	–0.07	0.09	0.04	Crowded	2.17	0.13	–0.05	0.06					

					Hyperventilation	0.60	–0.08	0.20	–0.05										

					Restless	–3.34	–0.05	0.15	0.06										

**Borderline features**																			

					Paranoia	0.68	–0.07	0.13	0.15	Fear abandonment	7.68	0.17**	–0.32*	0.16	Diff. being alone	38.61***	–0.08	0.19	0.37**

										Impulsivity	4.80	–0.09	–0.13	0.23*	Lonely	48.56***	–0.14	0.24	0.28**

															Good relations	–4.41	–0.15	0.01	0.44***

**Substance abuse**																			

										Craving	10.96*	0.00	1.05***	0.49***					

**Somatization and burn-out features**																			

															Pain	2.29	–0.01	0.00	–0.03

															Washed-out	0.43	0.03	0.02	0.09

															Empty	1.47	0.00	0.05	–0.03

Diff. saying no															Diff. indic. bound.	–1.06	0.64**	–0.54*	–0.01


*Note*. β_e_, β_d_, β_e*d_, β_l_ denote the *event* effect (lockdown for Patient 1 to 3 or family conflict for Patient 4), *day* trend before the event (with *day* centered around the event), the interaction between *event* and *day* predictors, and a *lagged* version of the modeled feature that refers to the previous day (to take into account serial dependencies), respectively. For each participant, we controlled for multiple testing using the false discovery procedure by Benjamini and Hochberg (1995). *p < .05, **p < .01, ***p < .001.

For Patient 1, we observe notable shifts in a substantial group of symptoms directly following the lockdown measures. While no symptom or emotion significantly increases or decreases before the lockdown was issued (β_d_), on the day of the lockdown, a significant increase is observed in levels of *experienced social pressure not to feel depressed* and *rumination, anxiety*, and in *difficulties saying no to people* (β_e_). This abrupt change is followed by significant increases over time in *depression, concentration problems, anxiety*, and *stress*, and decreases over time in *energy* and *social pressure not to feel depressed* after the lockdown (β_e*d_). These effects are observed after controlling for the self-predictiveness (β_l_) of day-to-day emotion or symptom intensities (which was significant for all features, except *energy*, feeling *stressed* and *relaxed*, and feeling like *going outside*).

Similarly, for Patient 2, we do not observe significant trends in symptom or emotion intensity before a lockdown took place (β_d_). However, in contrast to Patient 1, also after the lockdown all mood and symptoms of Patient 2 remain completely unaffected, showing no remarkable sudden shifts (β_e_) or gradual aggravations (β_e*d_) after the lockdown was issued. Furthermore, daily symptom or emotion intensities were never significantly auto-correlated (β_l_).

Next, Patient 3 presents evidence for symptom-specific worsening: While we observe minimal gradual increases over time in symptom and emotion levels before the lockdown began (β_d_; e.g., feeling more *down* or *anxious*, more *rumination* and *suppression*, but no changes in, for example, *anhedonia, self-esteem* or *impulsivity*), only her *craving* levels for amphetamines significantly increased on the day of the lockdown (β_e_), and increased further over time (β_e*d_) after the lockdown. For all other complaints, critical changes following the lockdown remained absent (e.g., feeling *down* or *anxious*). Similar to Patient 1, all Patient 3’s assessed features were self-predictive, except for experiencing *fear* of abandonment and *crowdedness* (β_l_).

Finally, for Patient 4, clear shifts in several emotions and symptoms are observed, similar to those observed for Patient 1 and 3. However, these changes occur directly following an unrelated family dispute that precedes the lockdown measures (February 14^th^). On the day of the dispute, clear upward shifts in *loneliness* and *difficulties being alone*, and downward shifts in *cheerfulness* and feelings of being *relaxed* are observed (β_e_) that remain stable in the time period following the family conflict (i.e., non-significant β’s_e*d_). In contrast, Patient 4’s *difficulties indicating boundaries* gradually decreased before the family conflict (β_d_) and significantly increased over time thereafter (β_e*d_). Taken together, these changes mirror some of the changes in symptom and emotion trajectories observed in other patients in response to the first COVID-19 lockdown in Belgium. Finally, only Patient 4’s daily experienced *difficulties being alone, loneliness* levels, and *good relations* with others were self-predictive (β_l_).

## Discussion

Taken together, these empirical time series data show four different trajectories in the short-term psychological responsiveness to the COVID-19 pandemic and associated measures taken by the Belgian government during the first wave in March 2020 in at-risk populations with pre-existing psychiatric conditions. For some individuals, the start of the COVID-19 pandemic had detrimental effects on their mental health, showing a sudden steep decline in emotional well-being that coincides with the start of the first lockdown in Belgium. However, others show remarkable levels of resilience, with little to no change in well-being following the lockdown measures in the first wave, or limited impact on only some specific features. Moreover, in one patient significant changes in mental health were observed, however, not following the COVID-19 lockdown, but in response to a personally relevant family dispute that occurred a few weeks earlier, suggesting that for some, the impact of the start of the COVID-19 pandemic may not be more detrimental than the impact of other more common daily life stressors.

Although the current data only provide preliminary indications, these different mood and symptom trajectories suggest that, at least on the short-term, not all at-risk individuals suffer equally and inevitably from the COVID-19 pandemic. In combination with other resilience studies, this makes us believe that the COVID-19 crisis will not necessarily lead to a large-scale mental-health crisis that will unavoidably affect all people with a history of mental illness to a similar degree. Indeed, these differential patterns align with previous resilience research in at-risk populations to other stressors (e.g., Min et al., 2013; Waugh & Koster, 2015), and with other preliminary COVID-19 studies that reveal crucial person-level well-being moderators in other (non-clinical) populations (e.g., Grossman et al. 2021; Havnen et al., 2020; Paredes et al., 2021). However, with respect to other aspects, our work and other COVID-19 well-being studies also provide complementing perspectives. First, our study had critical baseline data about people’s well-being before the pandemic started, which is an essential benchmark to establish actual resilience (Bonanno & Burton, 2013). Second, the intensive and longitudinal nature of our ESM protocol allowed to detect fine-grained changes in well-being as the first wave of the pandemic gradually ensued, which is not possible in basic cross-sectional designs or single pre- versus post-assessments. Moreover, our idiosyncratic approach allowed us to detect changes in features that are directly relevant for a participant. In contrast, other COVID-19 well-being studies have sample sizes that are typically larger, allowing researchers to move beyond simple case evidence and to detect nomothetic regularities. Relatedly, other studies also generally provide more insight into the exact explanatory mechanisms or behavioral correlates that underlie differences in resilience (e.g., Grossman et al. 2021). Indeed, our work remains rather descriptive and does not provide possible explanations for the reason why some participants showed psychological deterioration and others did not. For example, in our study, no data was obtained on participants’ physical health condition, making it hard to assess whether differences in objective threat level could explain the current findings (i.e., we cannot rule out the possibility that Patient 1 and 3 have increased risk for COVID-19 infection or more severe disease complications upon actual contagion). Similarly, we did not assess individual differences in the general or situational coping strategies that would cultivate psychological resilience or symptom aggravation. Future COVID-19 well-being research could therefore benefit from integrating the strengths of both approaches, by adopting a more explicit focus on (changes in) the state-like or situational mechanisms or processes that underlie patients’ psychological well-being in response to this adversity. Here, previous resilience studies (e.g., Norris, Tracy & Galea, 2009) may provide both scientists and practitioners with useful leads for which situational coping strategies to investigate and implement (ranging from concrete behavioral strategies [e.g., trying to maintain a healthy day routine] to more cognitive approaches [e.g., practicing mindfulness and developing self-compassion]; Polizzi, Lynn, & Perry, 2020).

In sum, embedding our findings with the emerging COVID-19 well-being literature, we suspect that many at-risk individuals will likely show sufficient coping abilities to resiliently deal with the challenges they encounter as a result of the COVID-19 pandemic. We believe that emphasizing this resilience will empower people to overcome the difficulties they may experience as results of the pandemic (Silver, 2020). Nevertheless, for a significant group of vulnerable people, the impact of the pandemic could be more deleterious, and hence should not be underestimated. For those, timely and affordable professional help will be crucial.

## Additional Files

The additional files for this article can be found as follows:

10.5334/pb.1028.s1 Supplementary Materials 1. Full quantitative item list and exact phrasing.

10.5334/pb.1028.s2 Supplementary Materials 2. Complete graphical overview of symptom and emotion time series.
