# Supplementary material for: The Short-Term Psychological Impact of the COVID-19 Pandemic in Psychiatric Patients: Evidence for Differential Emotion and Symptom Trajectories in Belgium
Source: Psychol Belg. 2021 Jun 21;61(1):163–72. doi: 10.5334/pb.1028 (PMC8231474; doi:10.5334/pb.1028)
Supplement: Supplementary Materials 1. — Full quantitative item list and exact phrasing. [file pb-61-1-1028-s1.pdf]

Supplemental Materials 1 – Full quantitative item list and exact phrasing

Table 1. Full quantitative item list per participant, together with the exact phrasing of each question.

Items	Phrasing
**Patient 1**	
Depressed	How depressed do you feel right now?
Sad	How sad do you feel right now?
Cheerful	How cheerful do you feel right now?
Anhedonia	How difficult is it right now to enjoy activities?
Self-esteem	How much self-esteem do you have right now?
Rumination	Since the previous beep, to what extent have you been ruminating?
Suppression	Since the previous beep, to what extent have you suppressed your emotions?
Energy	How much energy do you have right now?
Conc. problems	How difficult is it concentrate right now?
Social pressure	How much social pressure do you experience not to feel depressed?
Anxious	How anxious do you feel right now?
Stressed	How stressed do you feel right now?
Relaxed	How relaxed do you feel right now?
Going outside	Since the previous beep, did you feel like going outside?
Diff. saying no	Since the previous beep, to what extent did you have difficulty saying “no”, even though you wanted to?
**Patient 2**	
Down	How down do you feel right now?
Guilt	How guilty do you feel right now?
Cheerful	How cheerful do you feel right now?
Self-esteem	How much self-esteem do you have right now?
Rumination	Since the previous beep, to what extent have you been ruminating?
Suppression	Since the previous beep, to what extent have you suppressed your emotions?
Tired	How tired do you feel right now?
Conc. problems	How difficult is it to concentrate right now?
Unease	How uncomfortable do your feel right now?
Anxious	How anxious do you feel right now?

Stressed	How stressed do you feel right now?
Relaxed	How relaxed do you feel right now?
Crowded	To what extent do you experience crowdedness around you right now?
Hyperventilation	To what extent did you suffer from hyperventilation?
Restless	How restless do you feel right now?
Paranoia	Since the previous beep, to what extent did you feel others were talking about you or watching you?
**Patient 3**	
Down	How down do you feel right now?
Frustrated	How frustrated do you feel right now?
Cheerful	How cheerful do you feel right now?
Anhedonia	How difficult is it right now to enjoy activities?
Self-esteem	How much self-esteem do you have right now?
Rumination	Since the previous beep, to what extent have you been ruminating?
Suppression	Since the previous beep, to what extent have you suppressed your emotions?
Energy	How much energy do you have right now?
Conc. problems	How difficult is it concentrate right now?
Anxious	How anxious do you feel right now?
Stressed	How stressed do you feel right now?
Relaxed	How relaxed do you feel right now?
Crowded	To what extent do you experience crowdedness around you right now?
Fear abandonment	To what extent are you afraid that others will abandon you right now?
Impulsivity	Since the previous beep, to what extent did you things without carefully thinking?
Craving	Since the previous beep, to what extent did you crave amphetamines?
**Patient 4**	
Anger	How angry do you feel right now?
Cheerful	How cheerful do you feel right now?
Energy	How much energy do you have right now?
Conc. problems	How difficult is it concentrate right now?
Relaxed	How relaxed do you feel right now?
Diff. being alone	How difficult do you find being alone right now?

Lonely	To what extent do you feel lonely right now?
Good relations	To what extent do you have good relations with other right now?
Pain	To what extent do you suffer from physical (pain)complaints right now?
Washed-out	How washed-out do you feel right now?
Empty	How empty do you feel right now?
Diff. indic. bound.	Since the previous beep, to what extent did you find it difficult to indicate your boundaries to others?

Note. Categorical and binary items are not presented, because their time series are difficult to visualize.
