# Supplementary material for: The Short-Term Psychological Impact of the COVID-19 Pandemic in Psychiatric Patients: Evidence for Differential Emotion and Symptom Trajectories in Belgium
Source: Psychol Belg. 2021 Jun 21;61(1):163–72. doi: 10.5334/pb.1028 (PMC8231474; doi:10.5334/pb.1028)
Supplement: Supplementary Materials 2. — Complete graphical overview of symptom and emotion time series. [file pb-61-1-1028-s2.pdf]

Supplemental Materials 2 – Complete graphical overview of symptom and emotion time series

All the time series of individual emotions and symptoms for each patient. All momentary states are rated on continuous slider scales ranging from 0 (*not at all*) to 100 (*very much*). In all graphs, the colored line represents the original time series data (averaged per day to get an indication of daily emotion and symptom fluctuations); the grey line shows the model-based predicted time-series, indicating fit. The black vertical line denotes the beginning of the lockdown period (issued on March 13th). For Patient 4, the green line depicts a self-reported family conflict (February 14th).

Patient 1

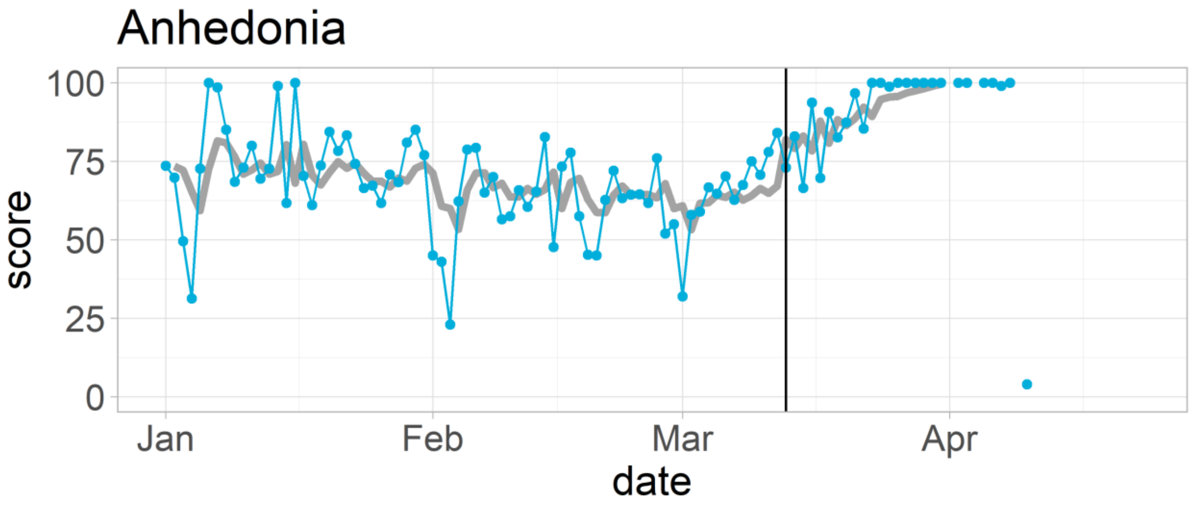

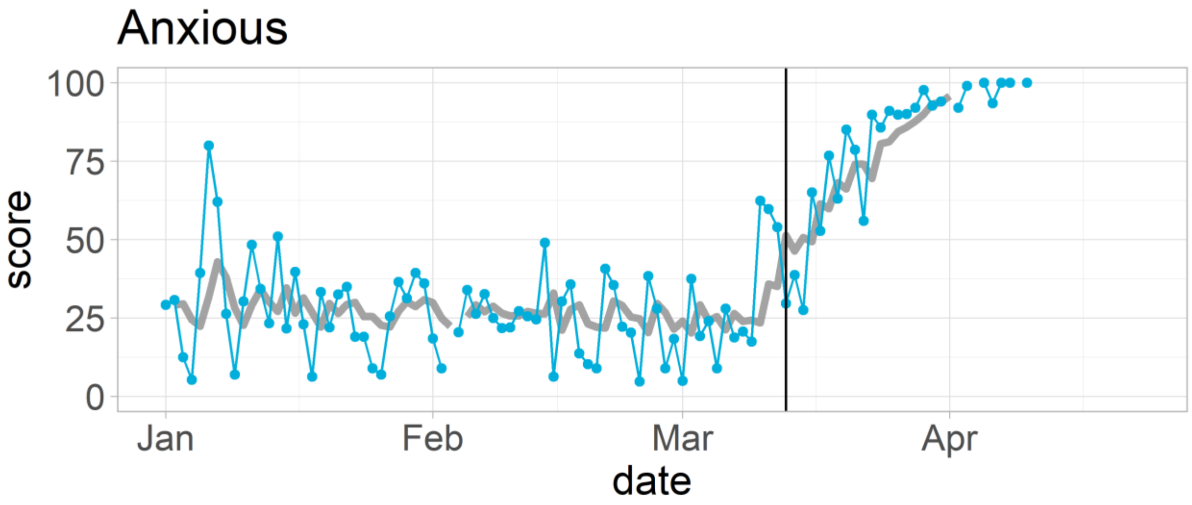

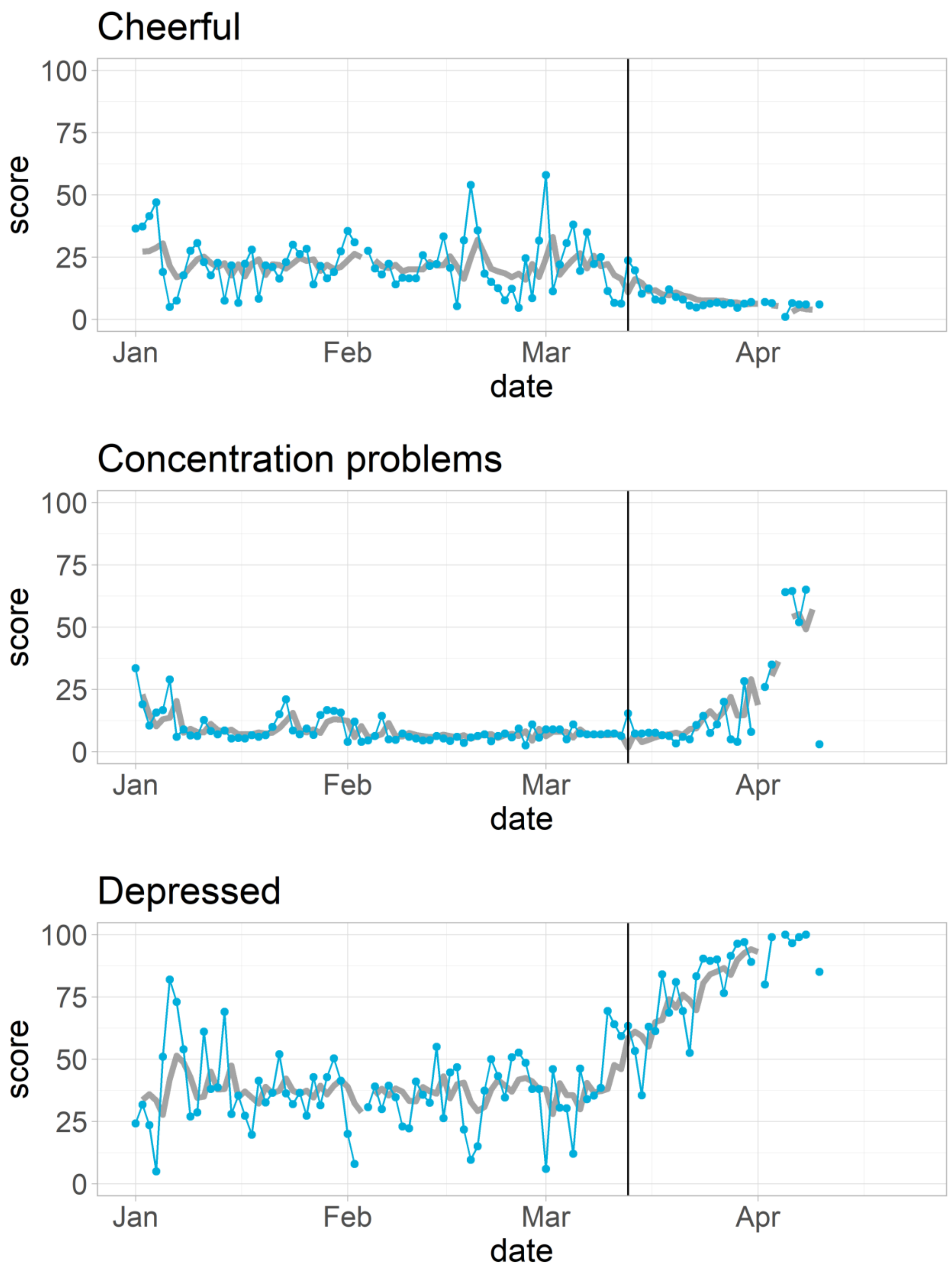

Cheerful
100
75
50
25
0
score
Jan
Feb
Mar
Apr
date
Concentration problems
100
75
50
25
0
score
Jan
Feb
Mar
Apr
date
Depressed
100
75
50
25
0
score
Jan
Feb
Mar
Apr
date

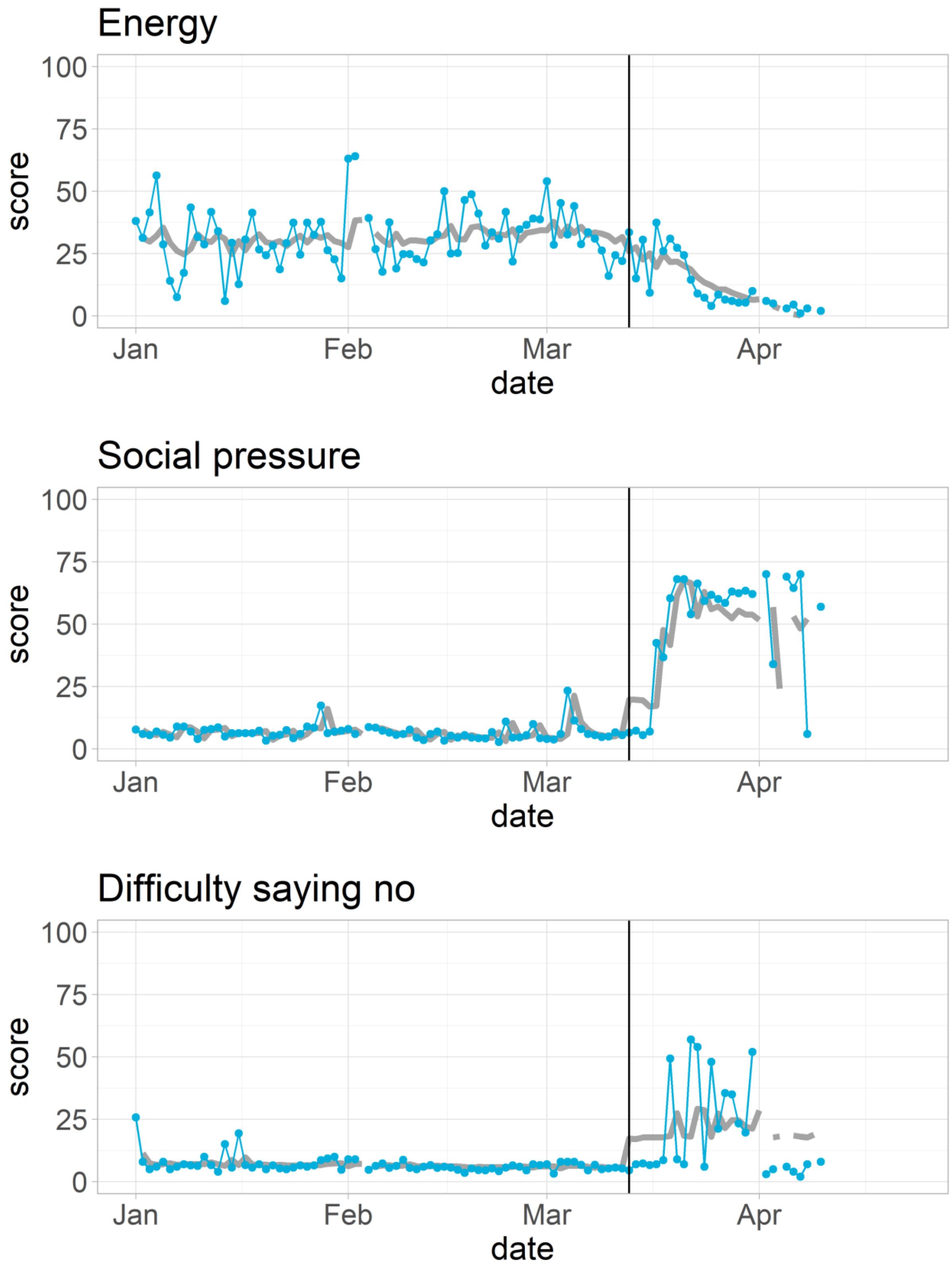
Energy
100
75
50
25
0
score
Jan
Feb
Mar
Apr
date
Social pressure
100
75
50
25
0
score
Jan
Feb
Mar
Apr
date
Difficulty saying no
100
75
50
25
0
score
Jan
Feb
Mar
Apr
date

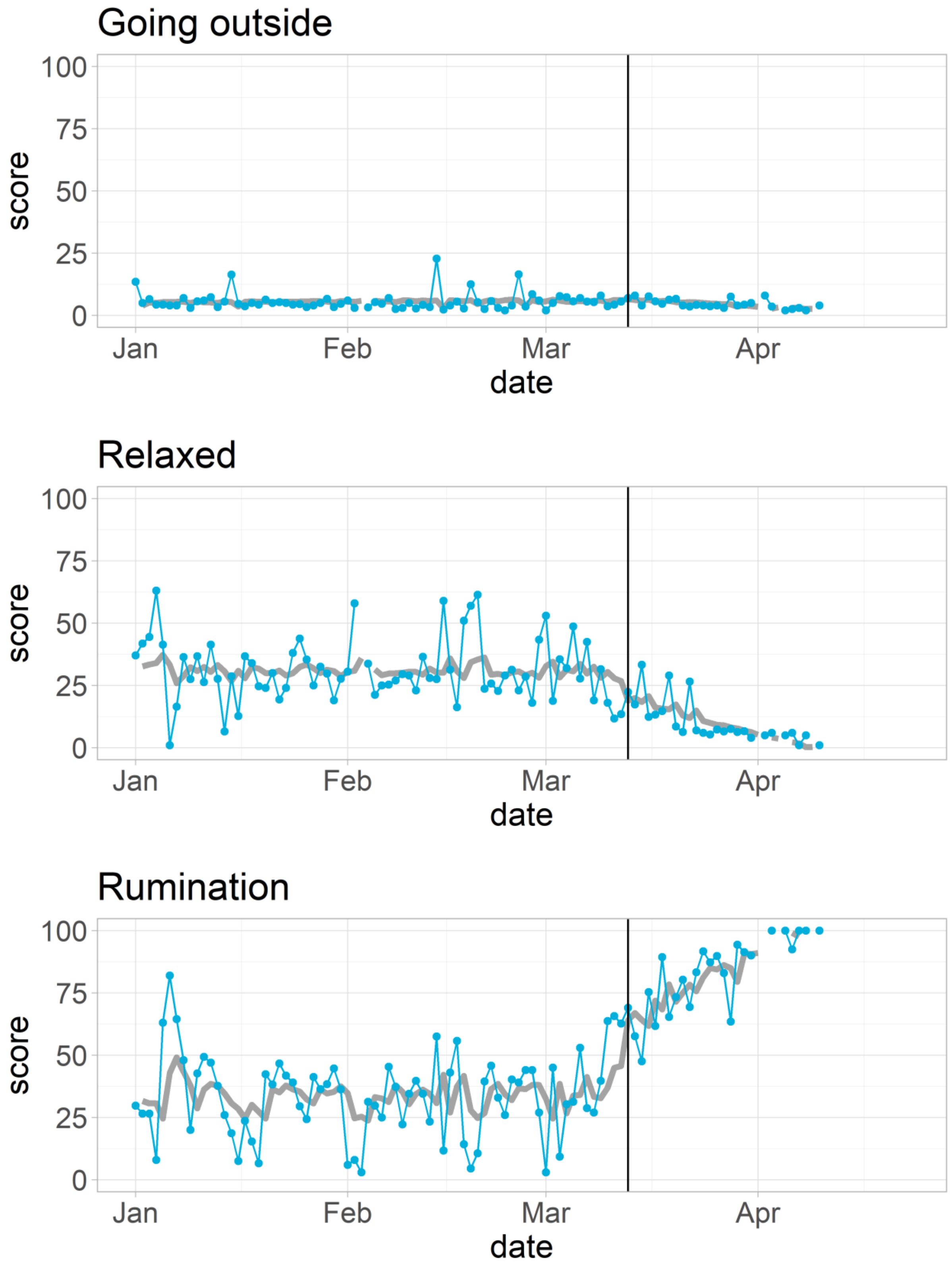

Going outside
100
75
50
25
0
score
Jan
Feb
Mar
Apr
date
Relaxed
100
75
50
25
0
score
Jan
Feb
Mar
Apr
date
Rumination
100
75
50
25
0
score
Jan
Feb
Mar
Apr
date

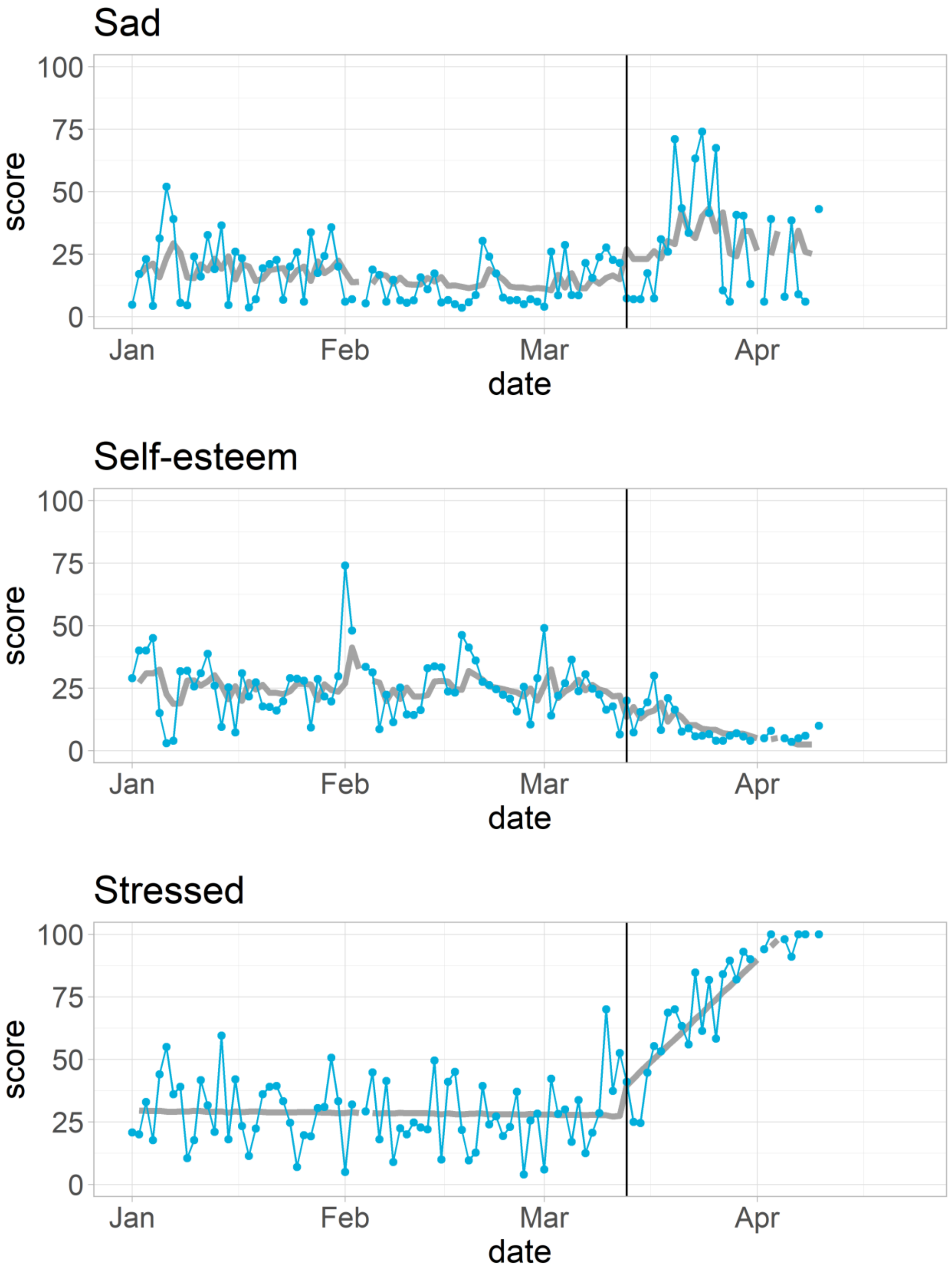

Sad
100
75
50
25
0
score
Jan
Feb
Mar
Apr
date
Self-esteem
100
75
50
25
0
score
Jan
Feb
Mar
Apr
date
Stressed
100
75
50
25
0
score
Jan
Feb
Mar
Apr
date

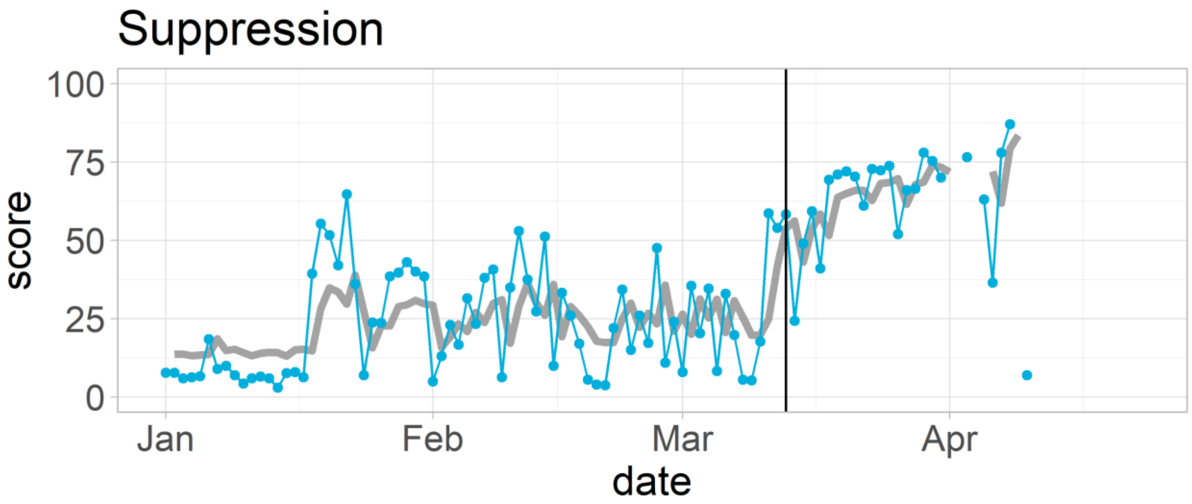

Suppression
score
100
75
50
25
0
Jan
Feb
Mar
Apr
date

Patient 2

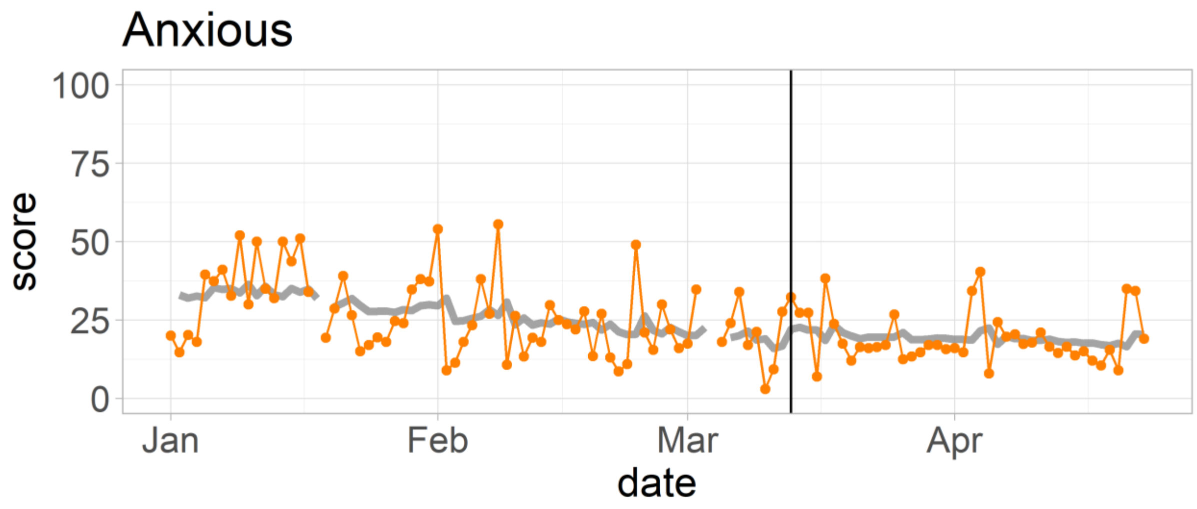

Anxious
score
100
75
50
25
0
Jan
Feb
Mar
Apr
date

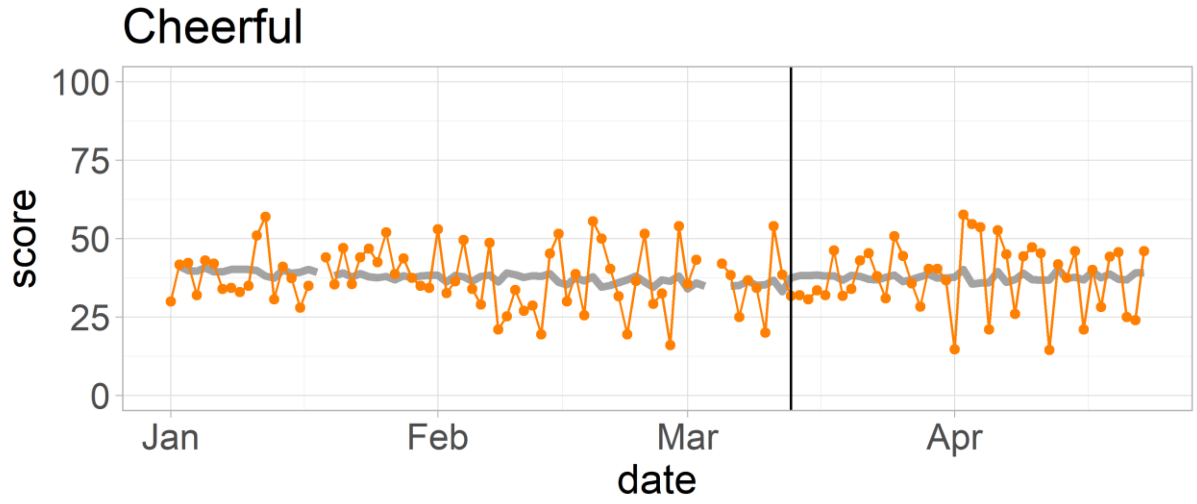

Cheerful
score
100
75
50
25
0
Jan
Feb
Mar
Apr
date

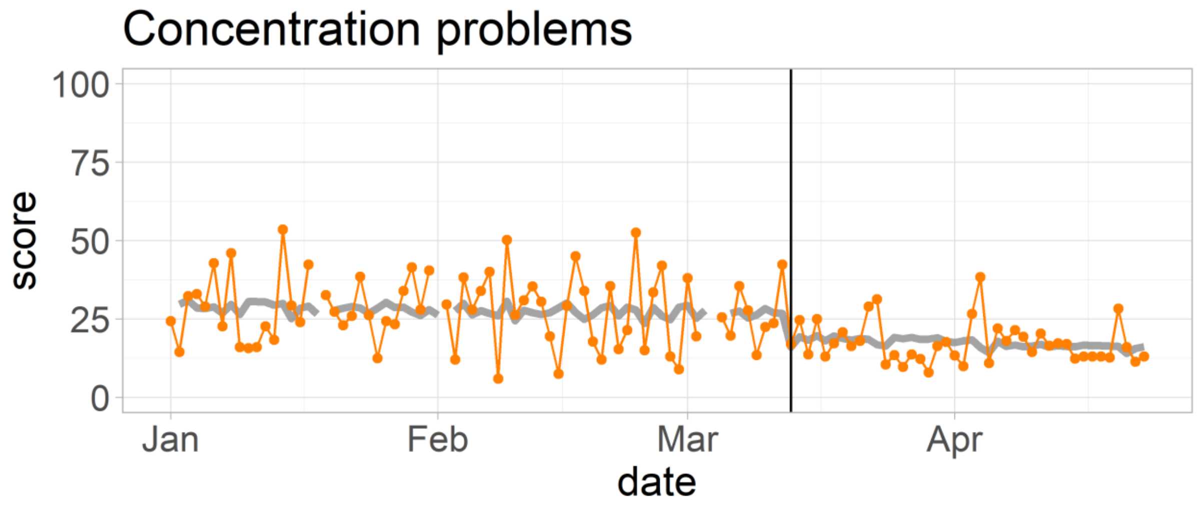

Concentration problems
score
100
75
50
25
0
Jan
Feb
Mar
Apr
date

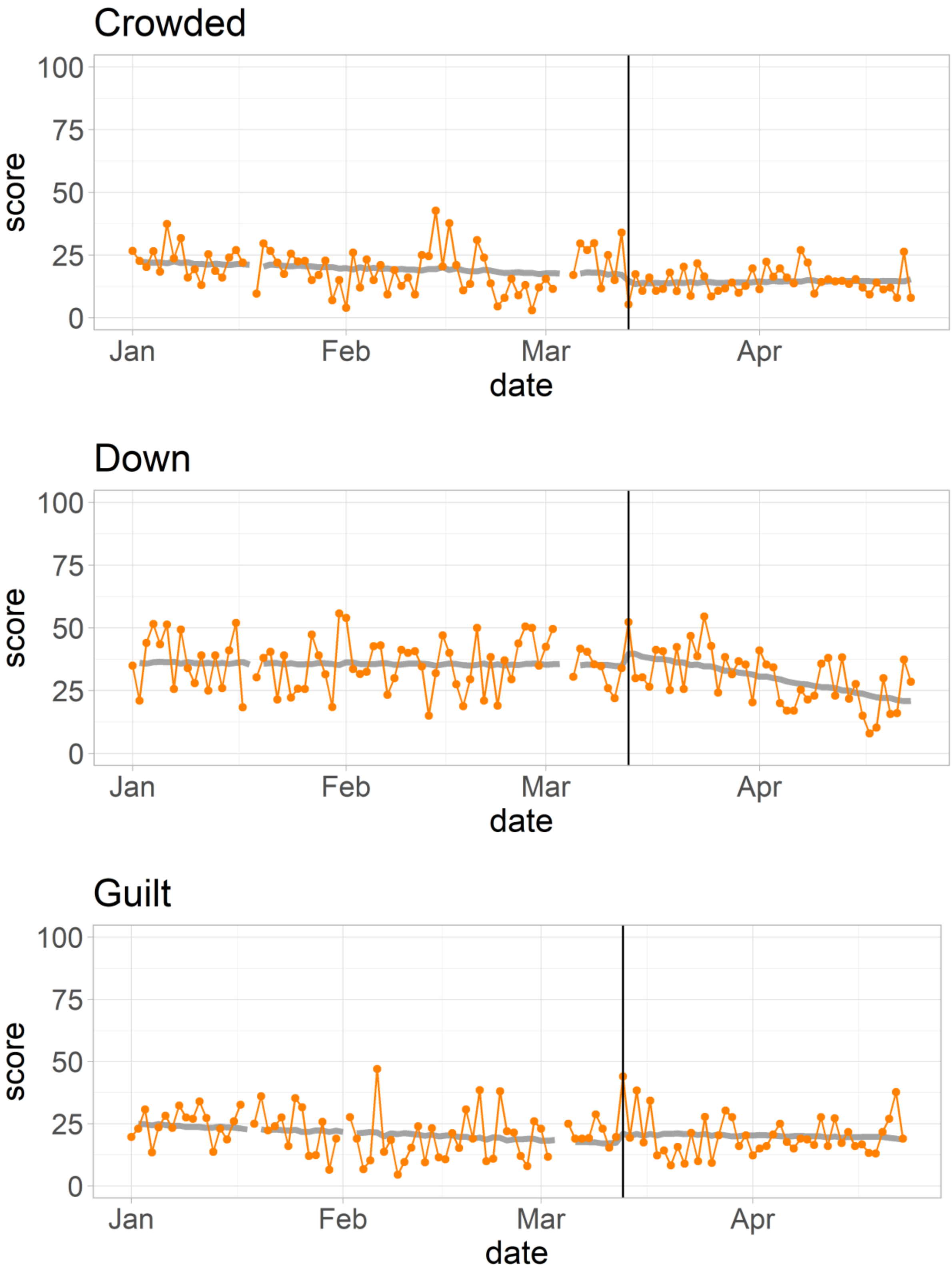
Crowded
score
100
75
50
25
0
Jan
Feb
Mar
Apr
date
Down
score
100
75
50
25
0
Jan
Feb
Mar
Apr
date
Guilt
score
100
75
50
25
0
Jan
Feb
Mar
Apr
date

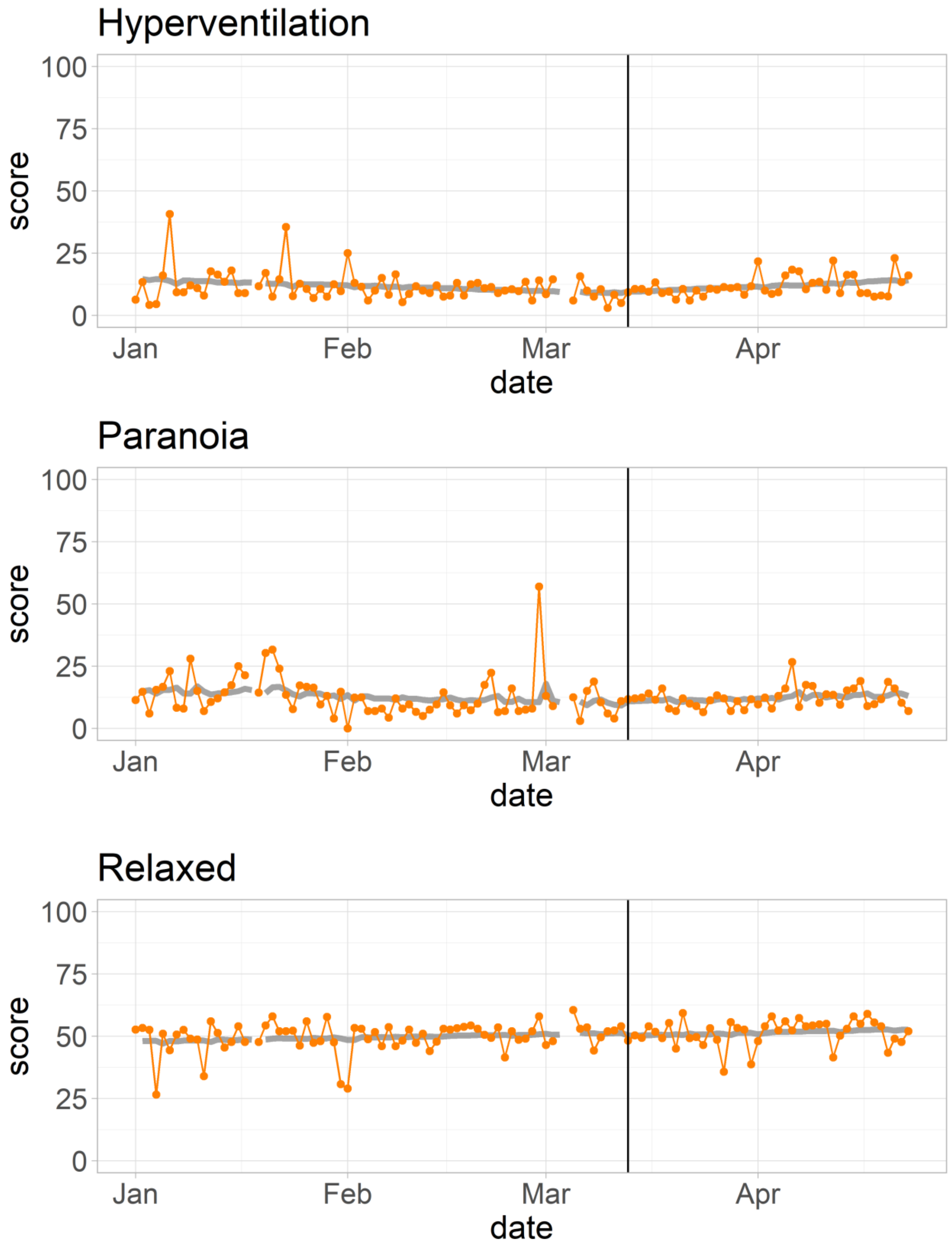

Hyperventilation
100
75
50
25
0
score
Jan
Feb
Mar
Apr
date
Paranoia
100
75
50
25
0
score
Jan
Feb
Mar
Apr
date
Relaxed
100
75
50
25
0
score
Jan
Feb
Mar
Apr
date

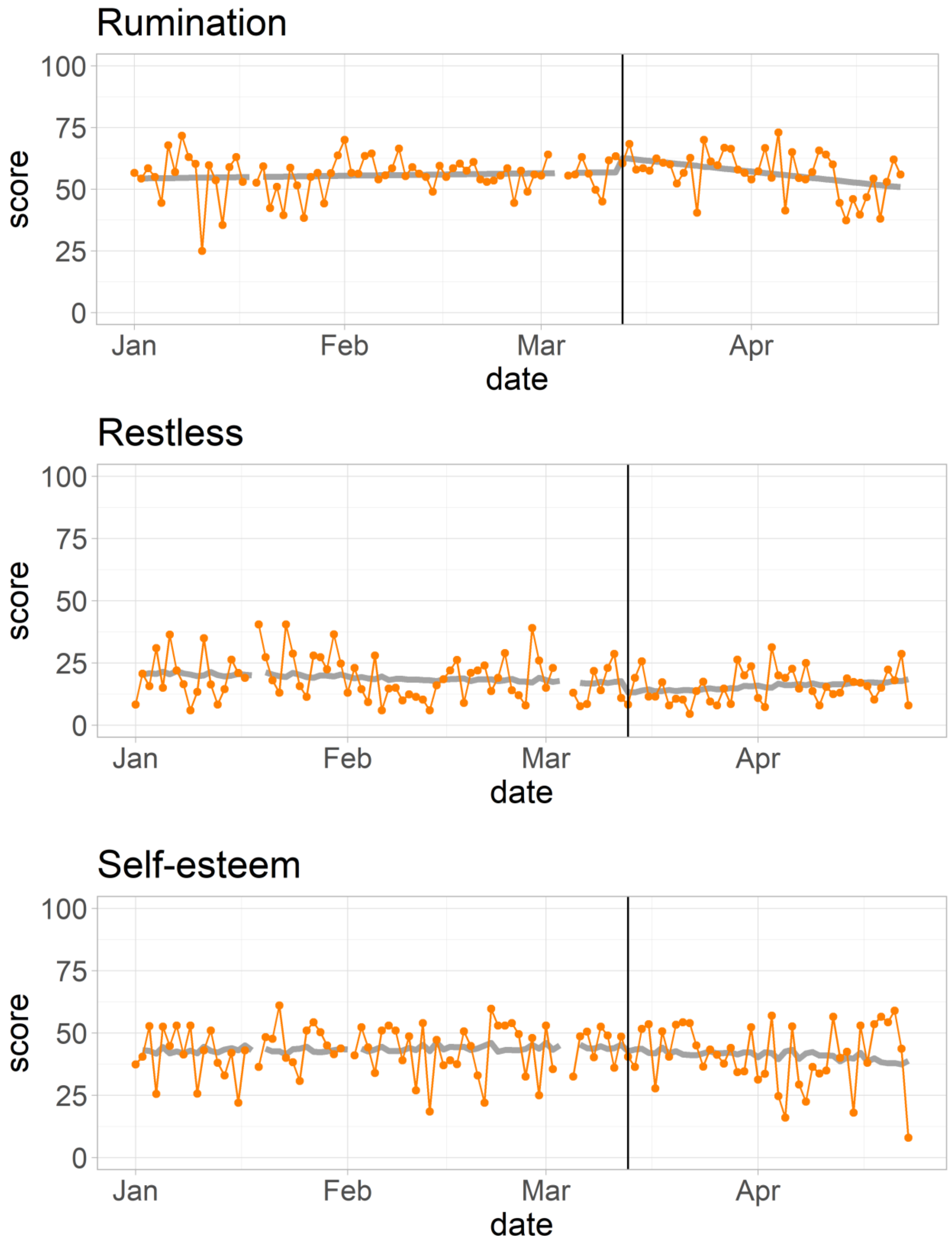
Rumination
100
75
50
25
0
score
Jan
Feb
Mar
Apr
date
Restless
100
75
50
25
0
score
Jan
Feb
Mar
Apr
date
Self-esteem
100
75
50
25
0
score
Jan
Feb
Mar
Apr
date

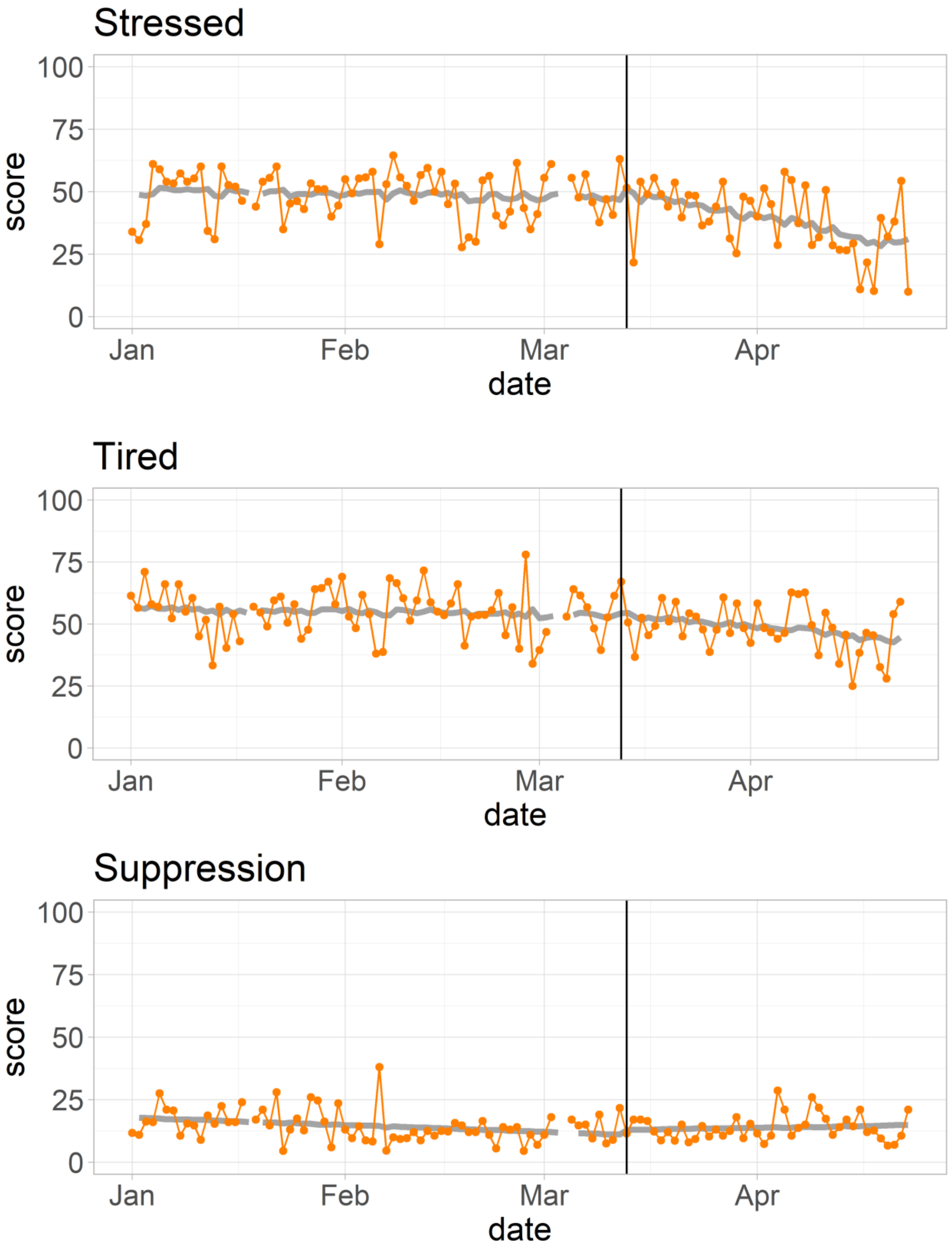
Stressed
score
100
75
50
25
0
Jan
Feb
Mar
Apr
date
Tired
score
100
75
50
25
0
Jan
Feb
Mar
Apr
date
Suppression
score
100
75
50
25
0
Jan
Feb
Mar
Apr
date

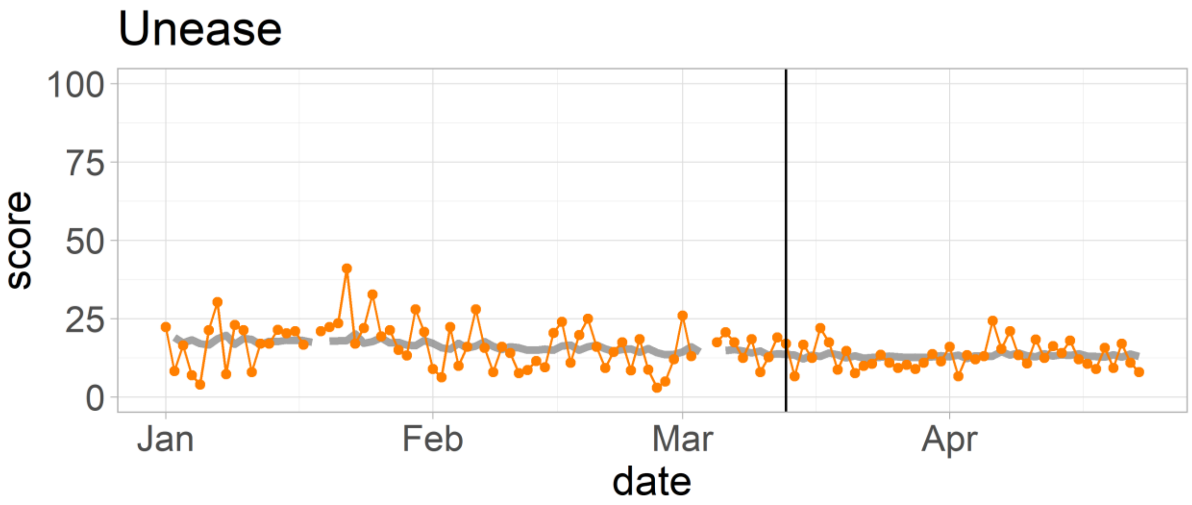
Unease
100
75
50
25
0
score
Jan
Feb
Mar
Apr
date

Patient 3

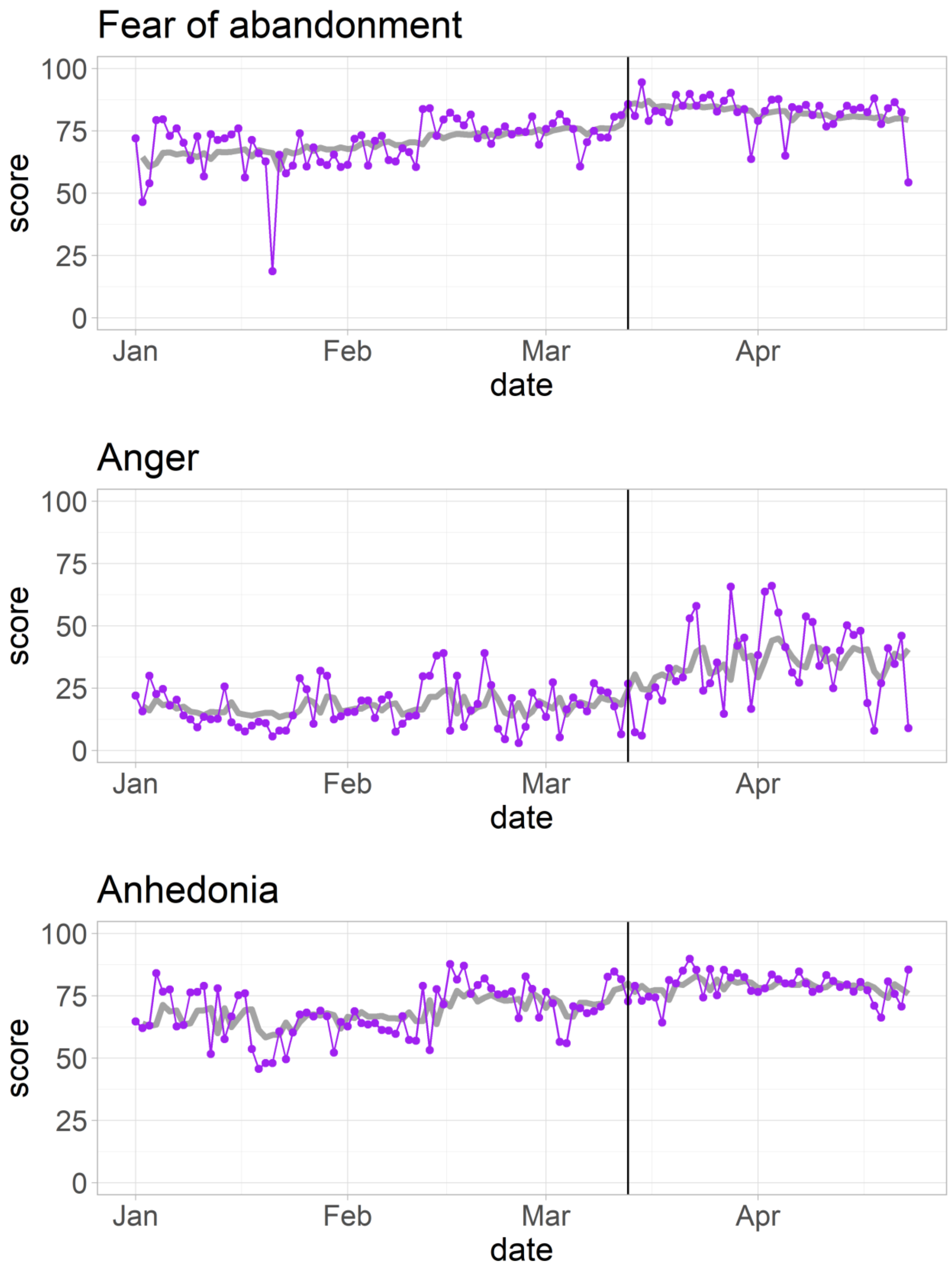

Fear of abandonment
100
75
50
25
0
score
Jan
Feb
Mar
Apr
date
Anger
100
75
50
25
0
score
Jan
Feb
Mar
Apr
date
Anhedonia
100
75
50
25
0
score
Jan
Feb
Mar
Apr
date

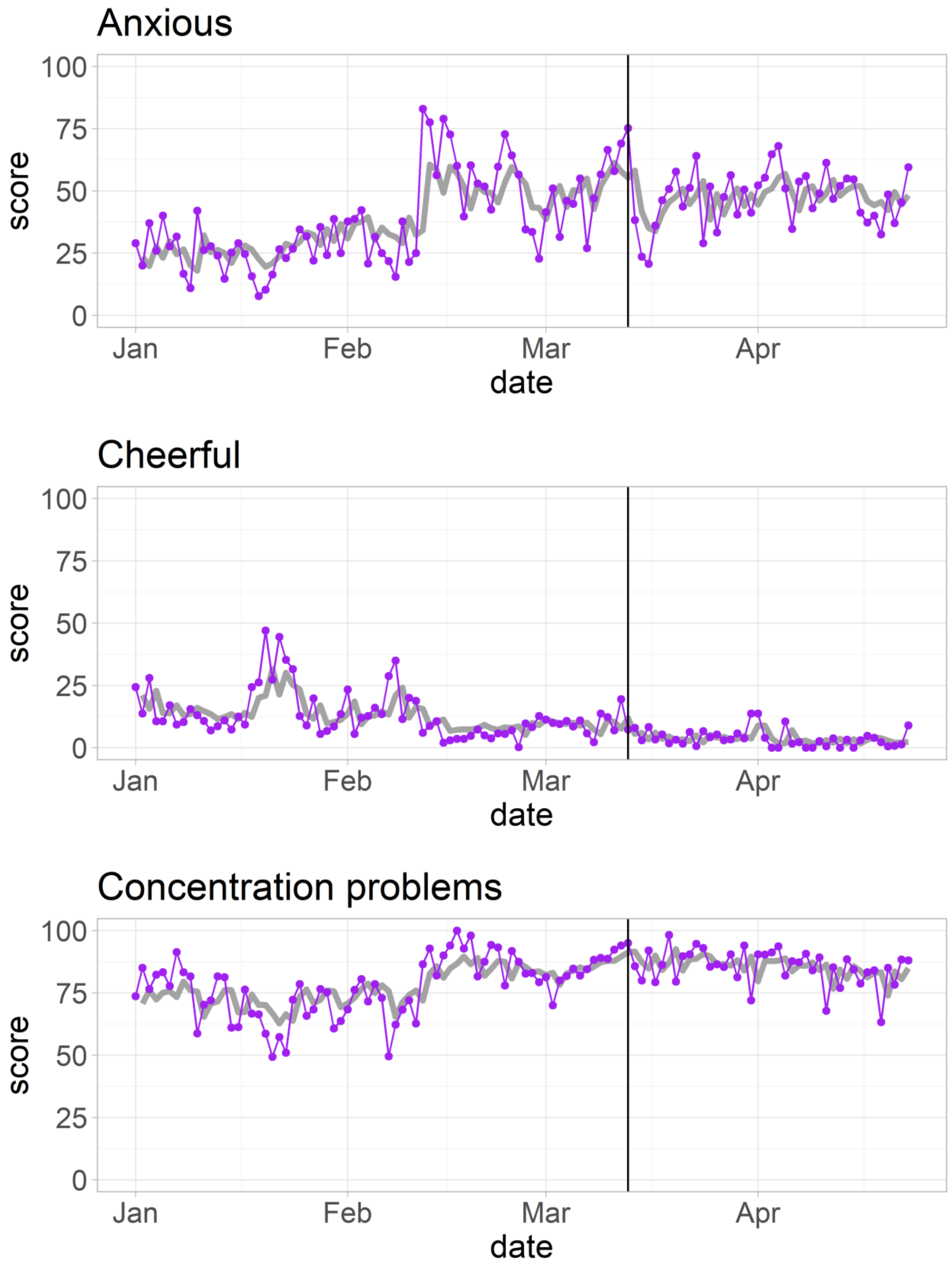
Anxious
Cheerful
Concentration problems
score
100
75
50
25
0
date
Jan
Feb
Mar
Apr

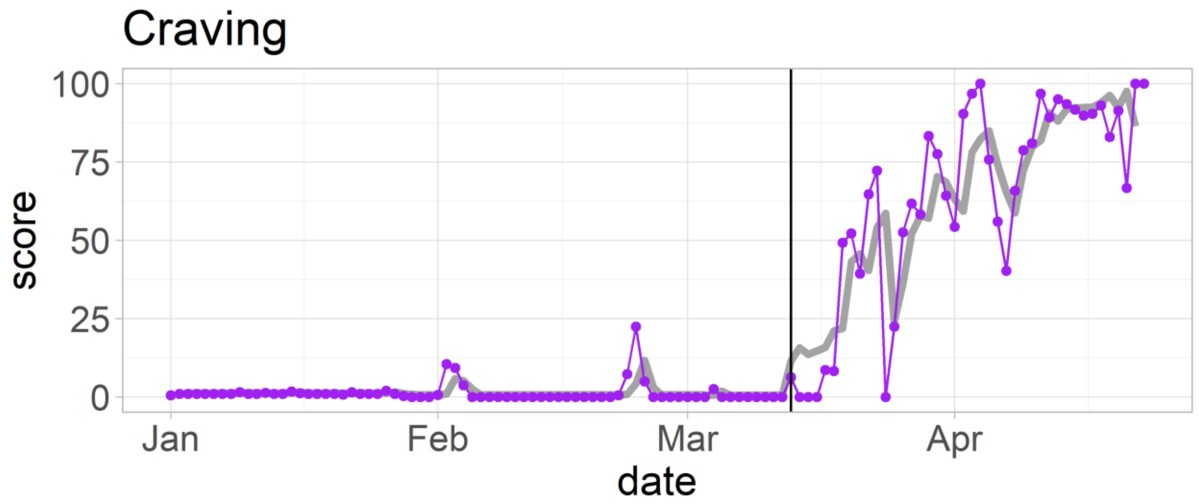

Craving
score
100
75
50
25
0
Jan
Feb
Mar
Apr
date

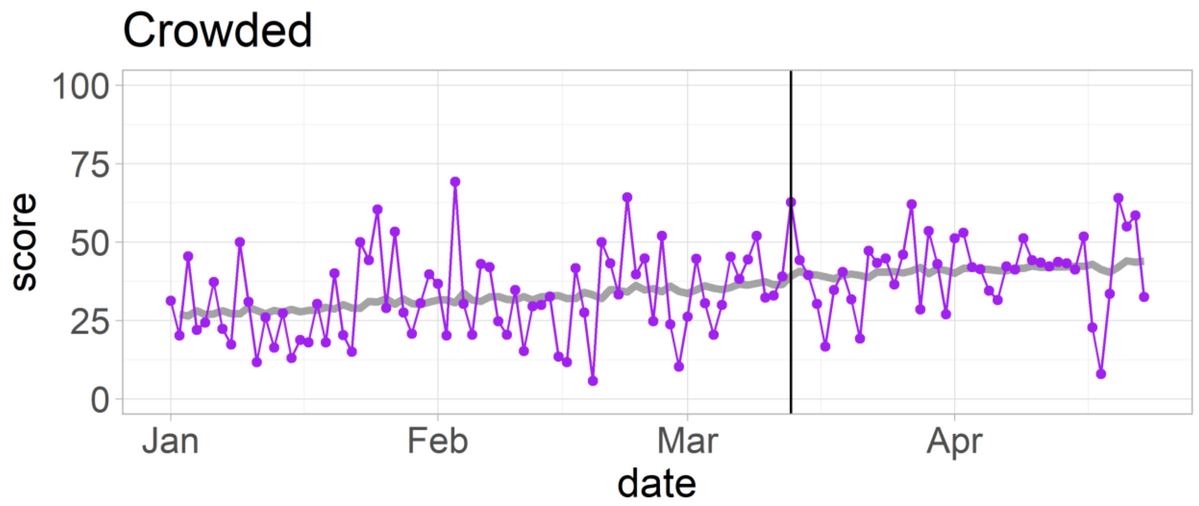

Crowded
score
100
75
50
25
0
Jan
Feb
Mar
Apr
date

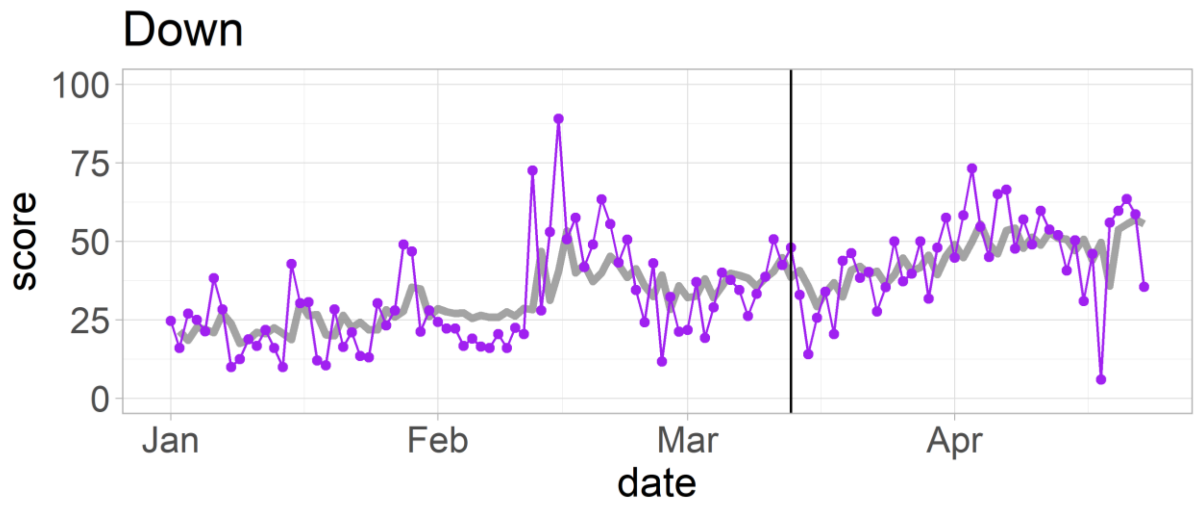

Down
score
100
75
50
25
0
Jan
Feb
Mar
Apr
date

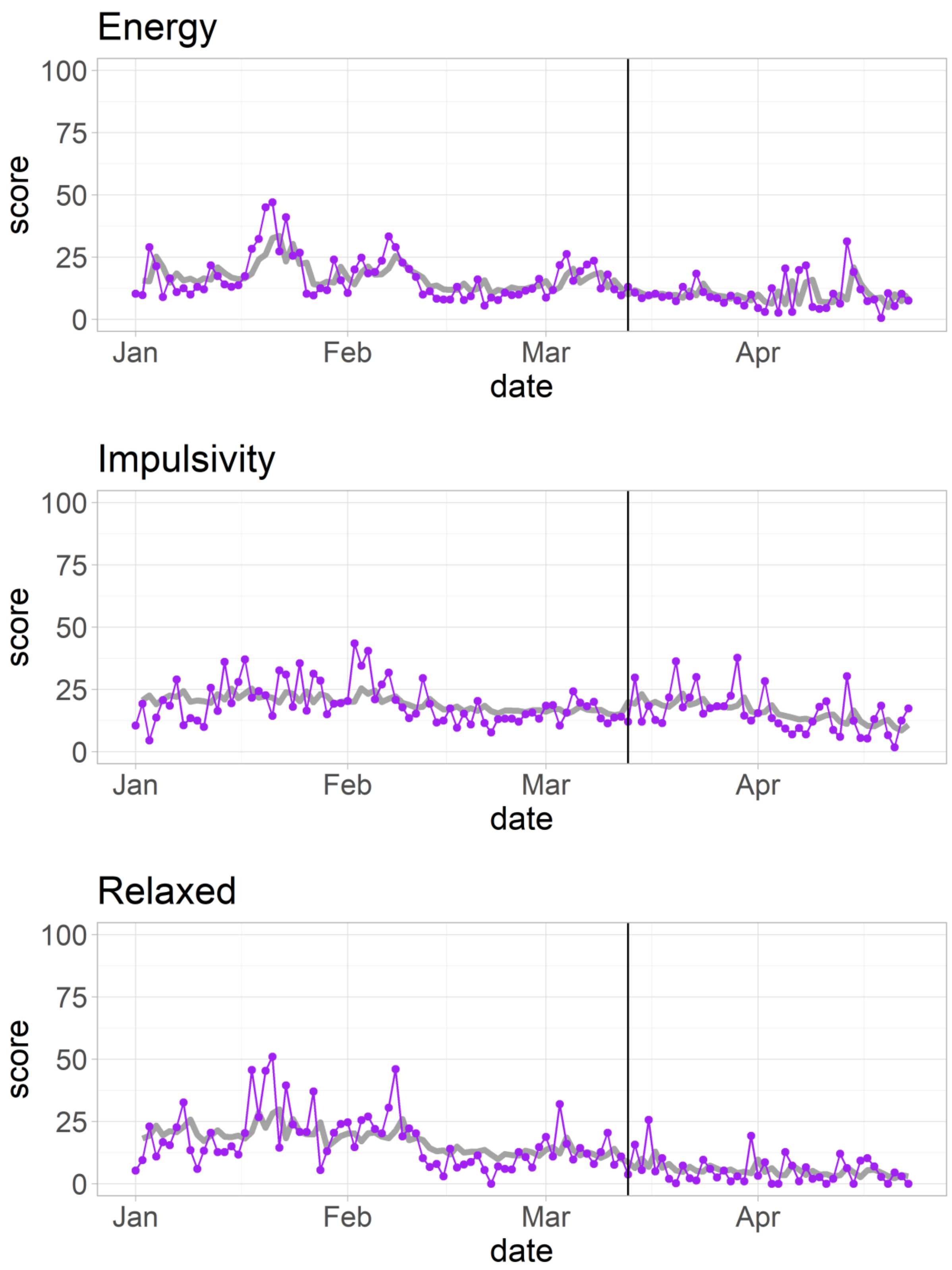
Energy
Impulsivity
Relaxed
score
100
75
50
25
0
Jan
Feb
Mar
Apr
date

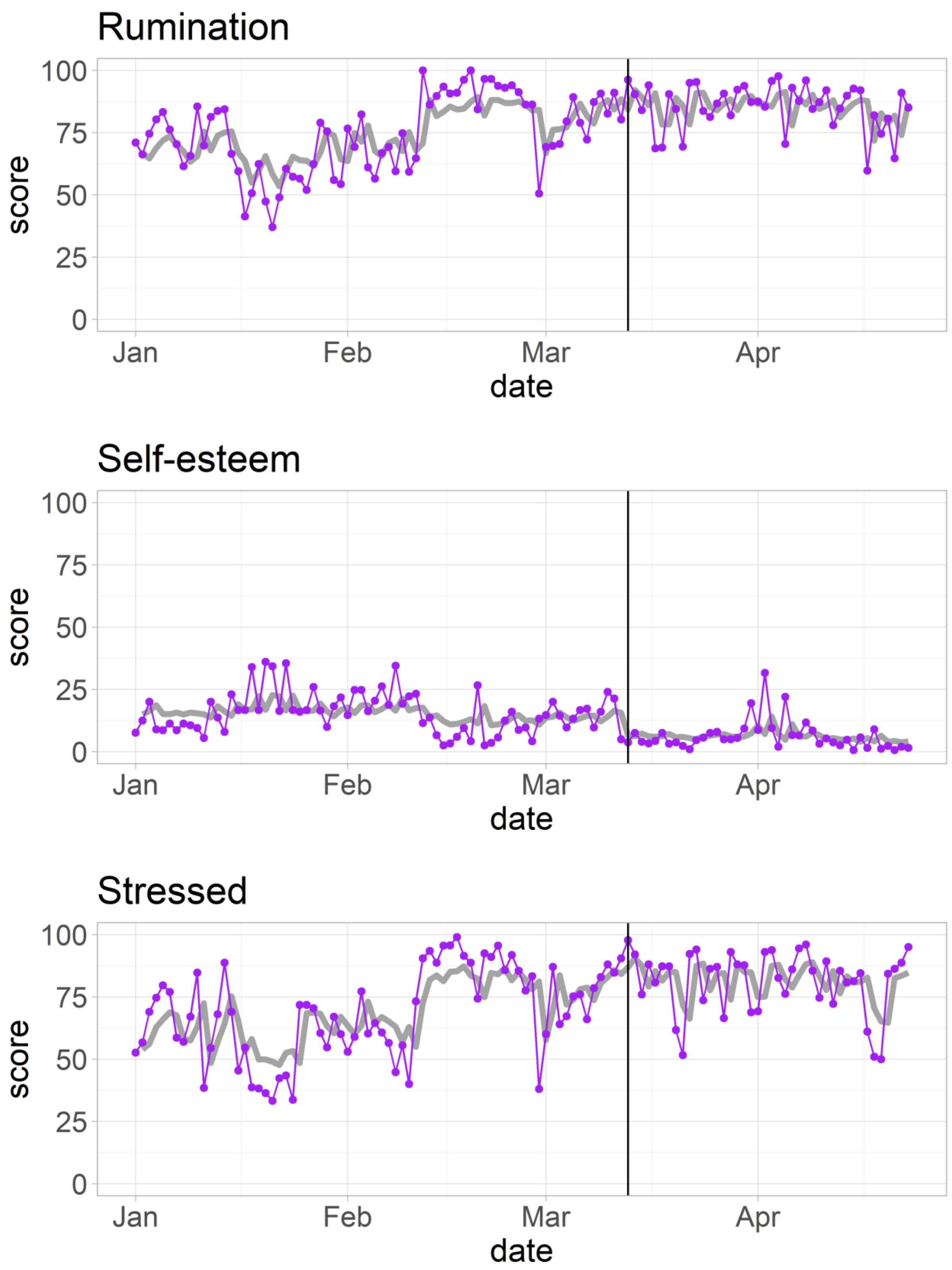
Rumination
100
75
50
25
0
score
Jan
Feb
Mar
Apr
date
Self-esteem
100
75
50
25
0
score
Jan
Feb
Mar
Apr
date
Stressed
100
75
50
25
0
score
Jan
Feb
Mar
Apr
date

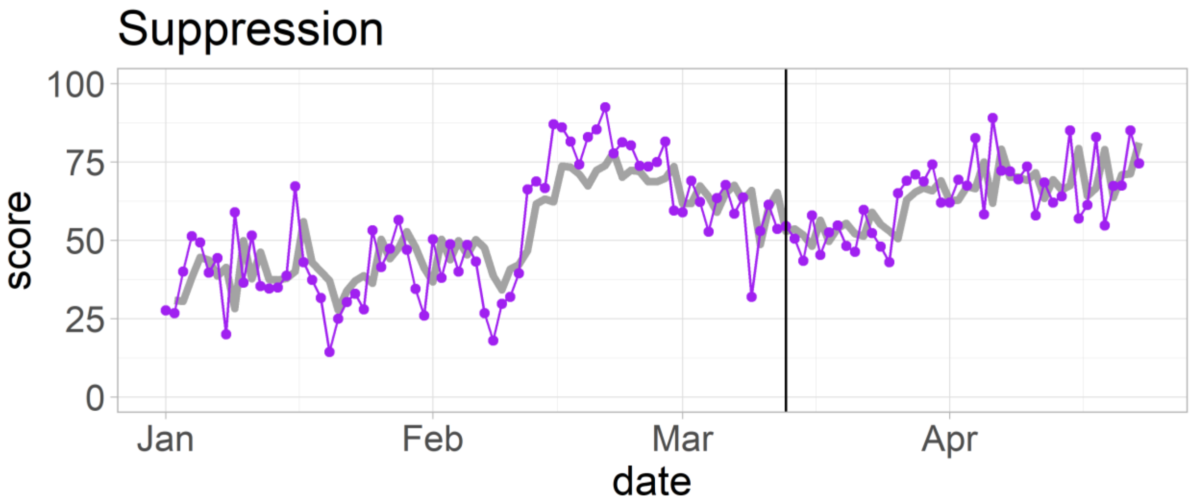

Suppression
score
100
75
50
25
0
Jan
Feb
Mar
Apr
date

Patient 4

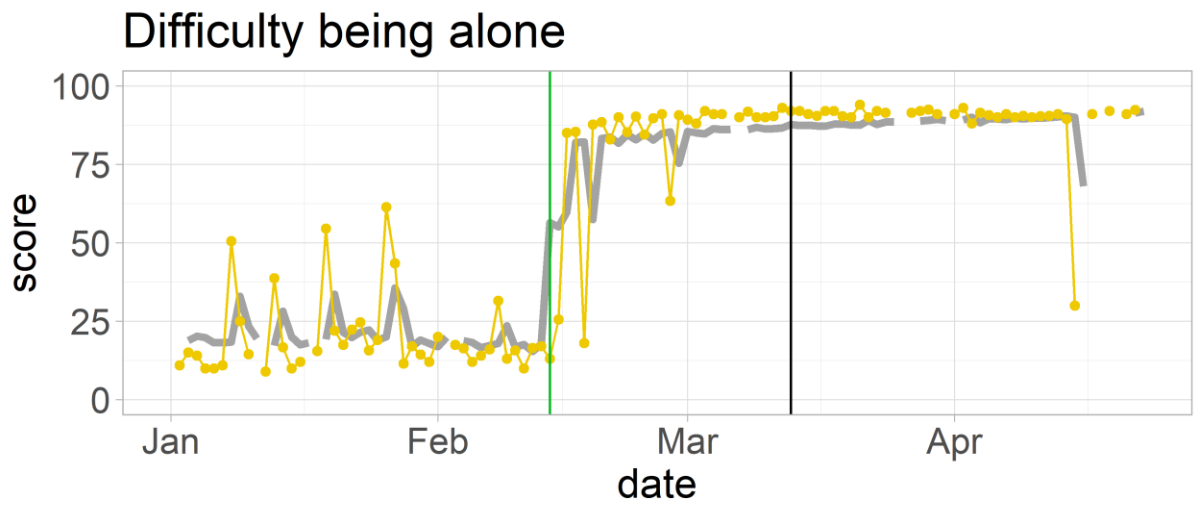
Difficulty being alone
score
100
75
50
25
0
Jan
Feb
Mar
Apr
date

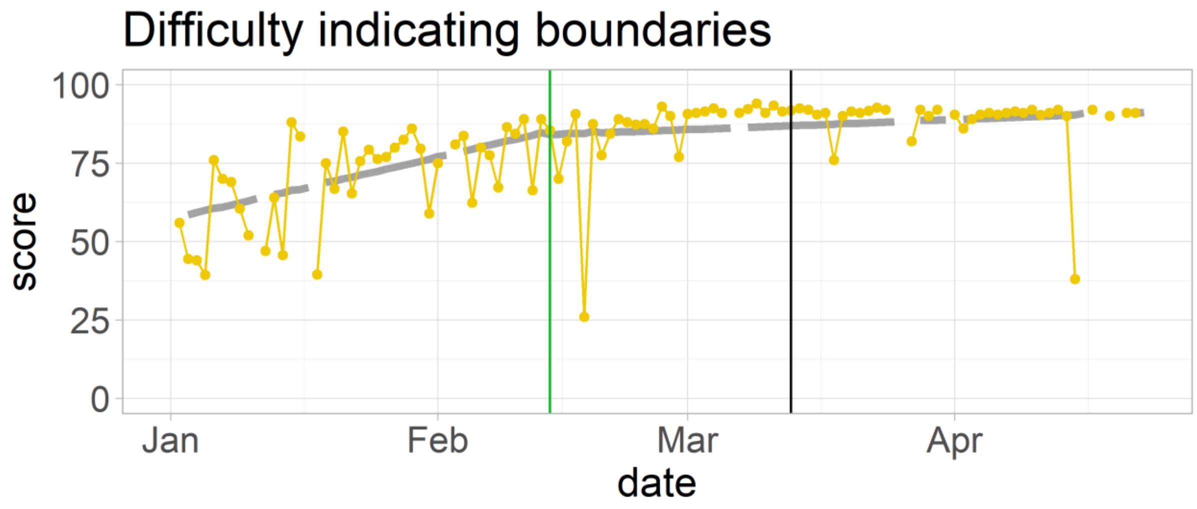
Difficulty indicating boundaries
score
100
75
50
25
0
Jan
Feb
Mar
Apr
date

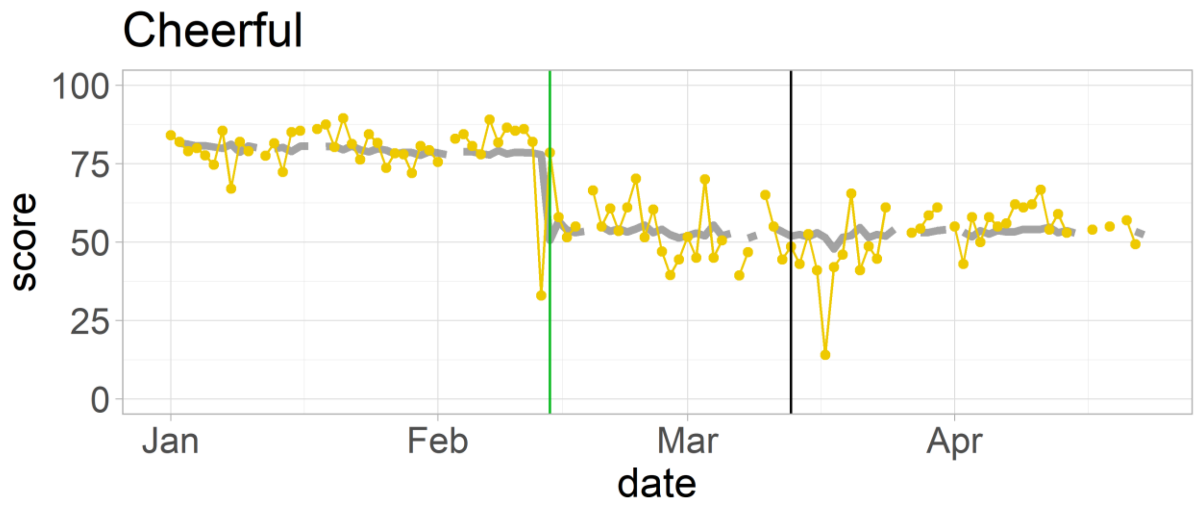
Cheerful
score
100
75
50
25
0
Jan
Feb
Mar
Apr
date

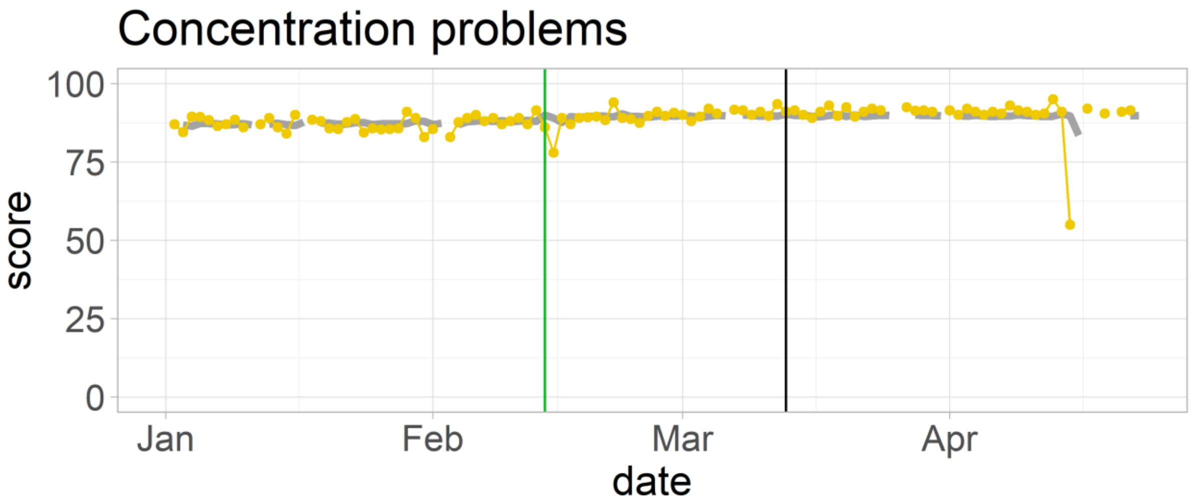
Concentration problems
score
100
75
50
25
0
Jan
Feb
Mar
Apr
date

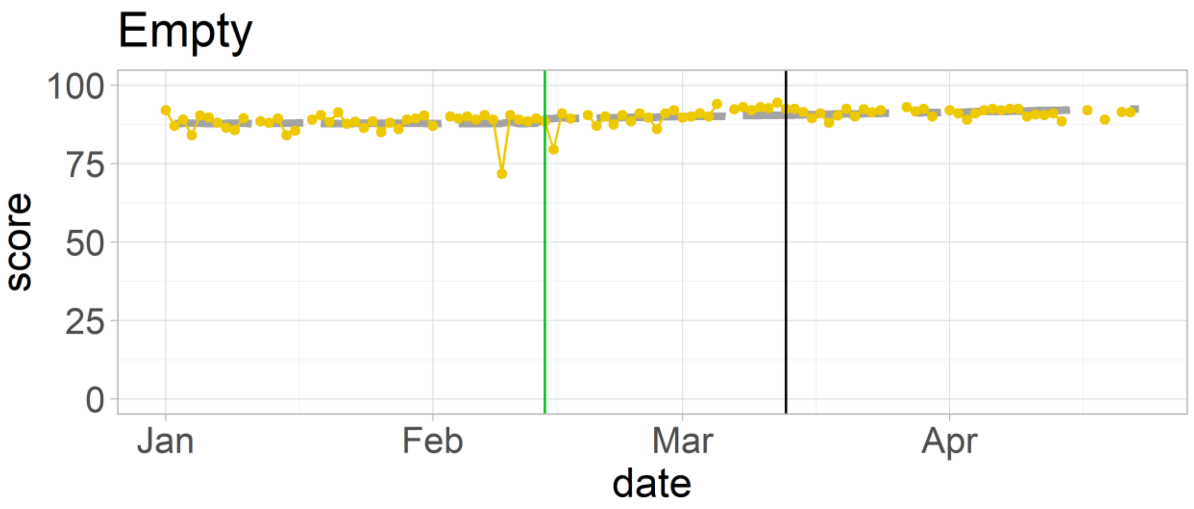
Empty
score
100
75
50
25
0
Jan
Feb
Mar
Apr
date

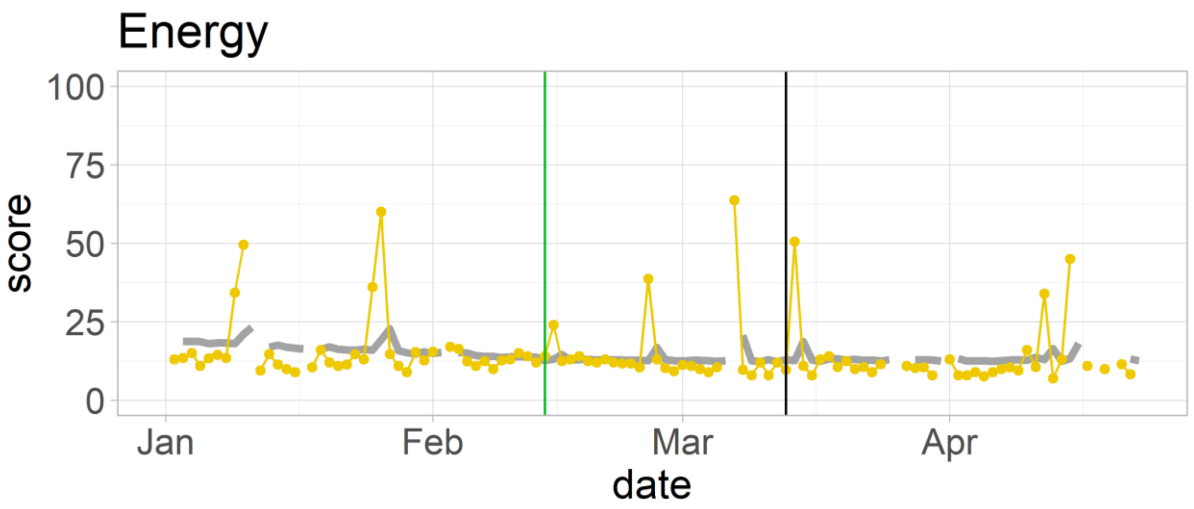
Energy
score
100
75
50
25
0
Jan
Feb
Mar
Apr
date

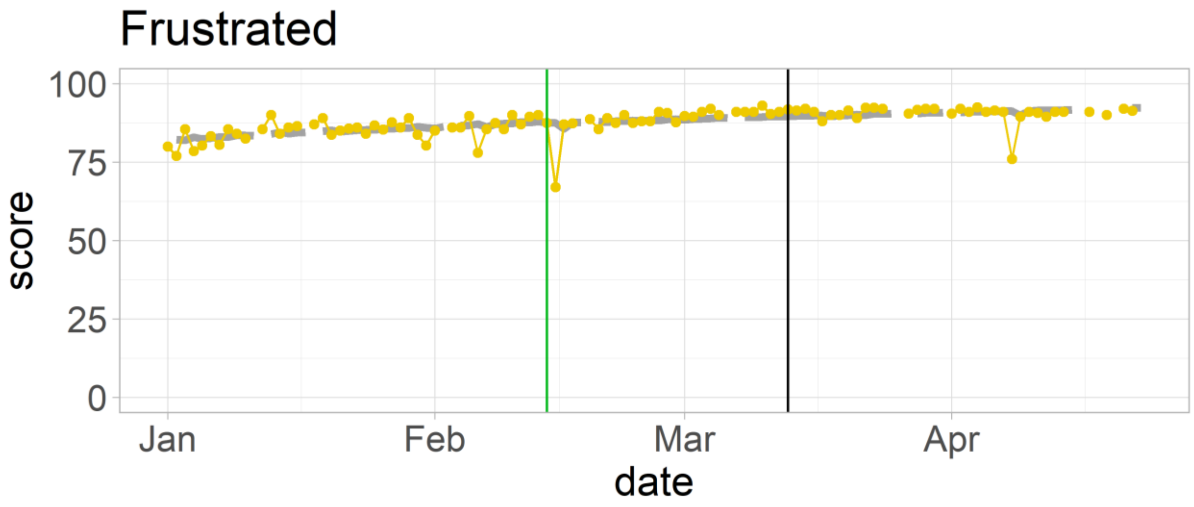
Frustrated
score
100
75
50
25
0
Jan
Feb
Mar
Apr
date

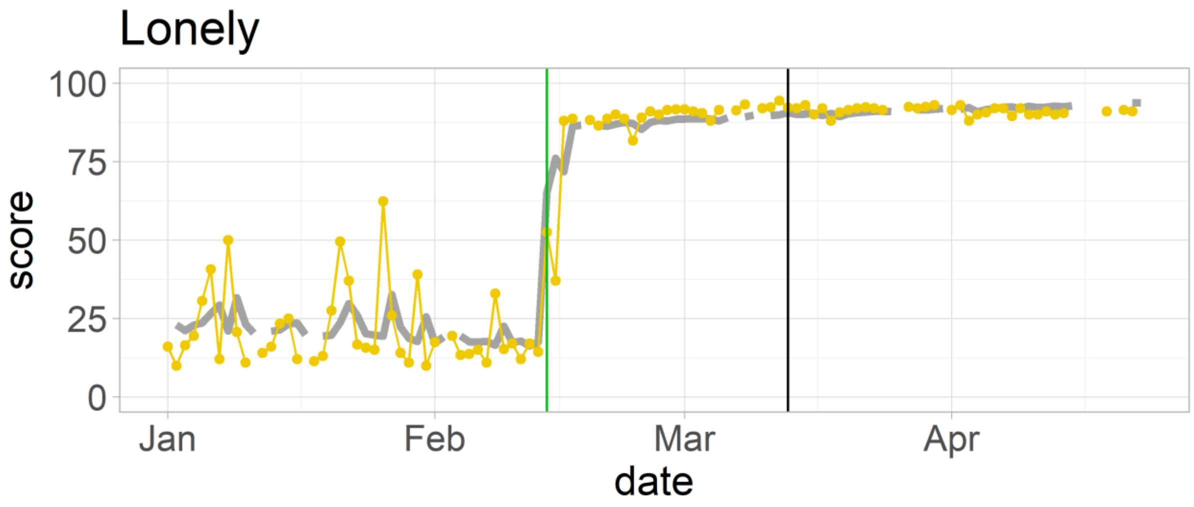
Lonely
score
100
75
50
25
0
Jan
Feb
Mar
Apr
date

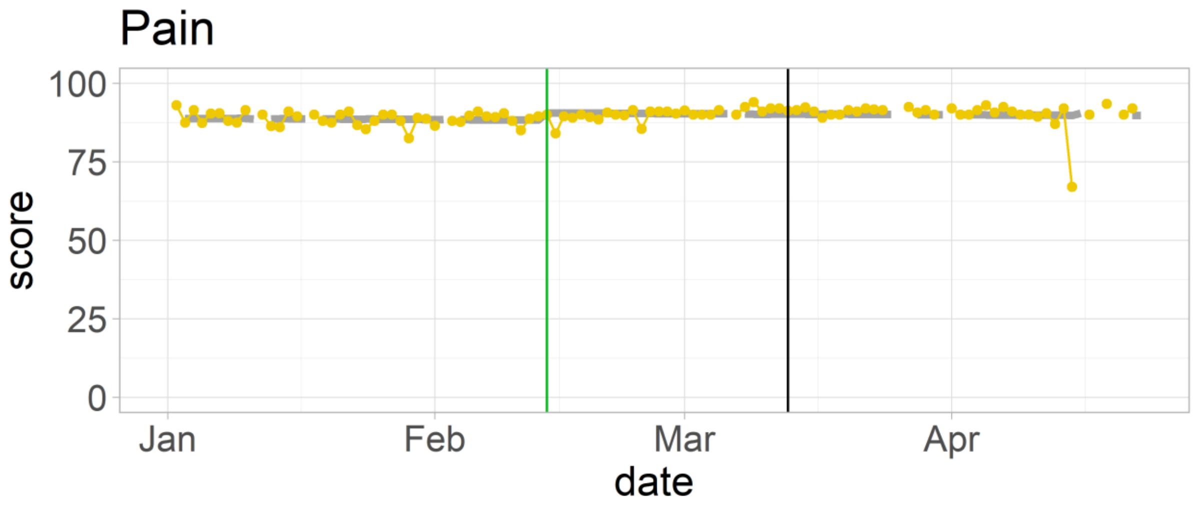
Pain
score
100
75
50
25
0
Jan
Feb
Mar
Apr
date

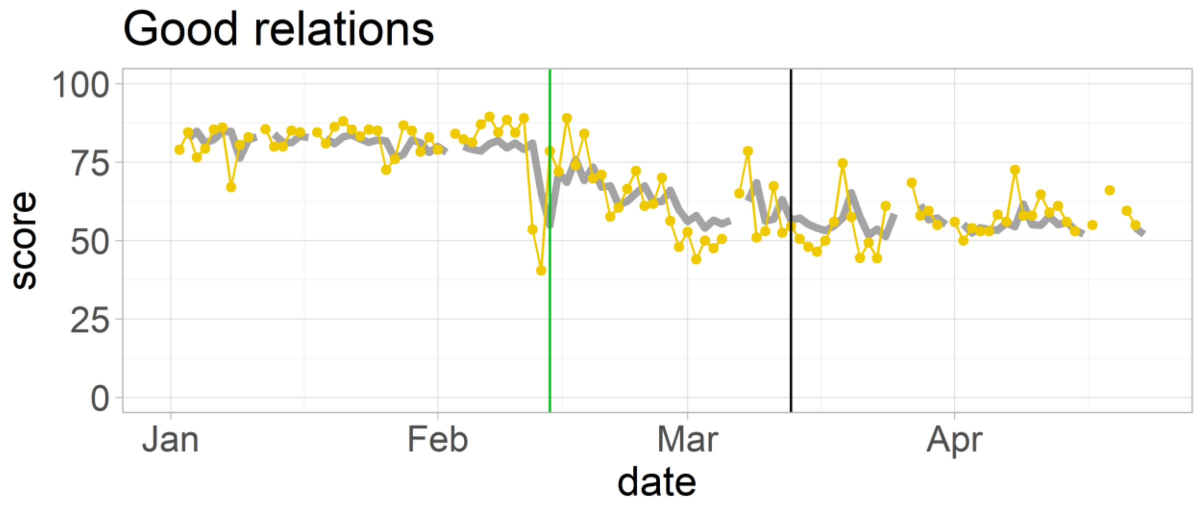
Good relations
100
75
50
25
0
score
Jan
Feb
Mar
Apr
date

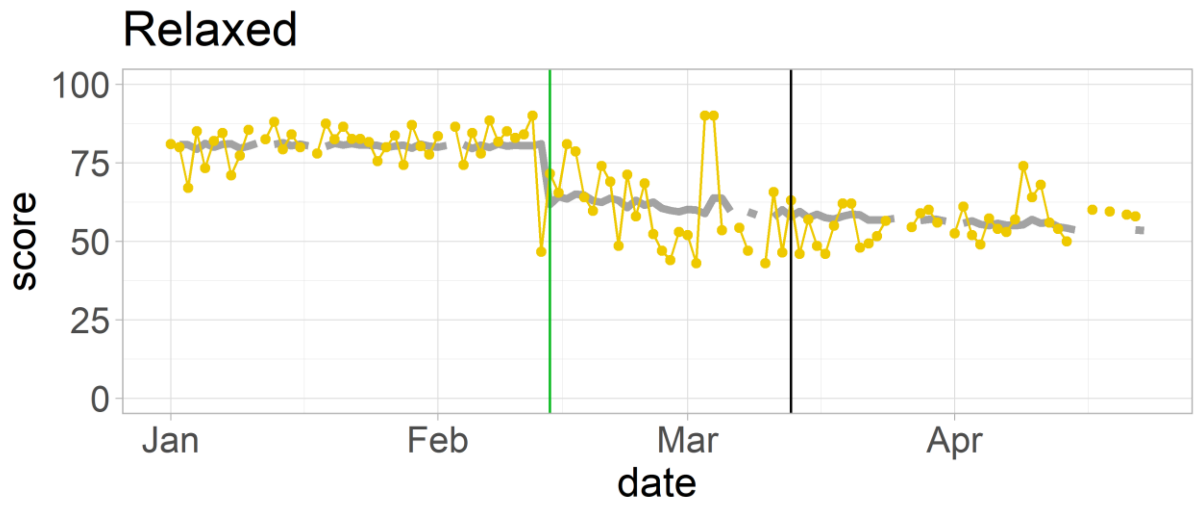
Relaxed
100
75
50
25
0
score
Jan
Feb
Mar
Apr
date

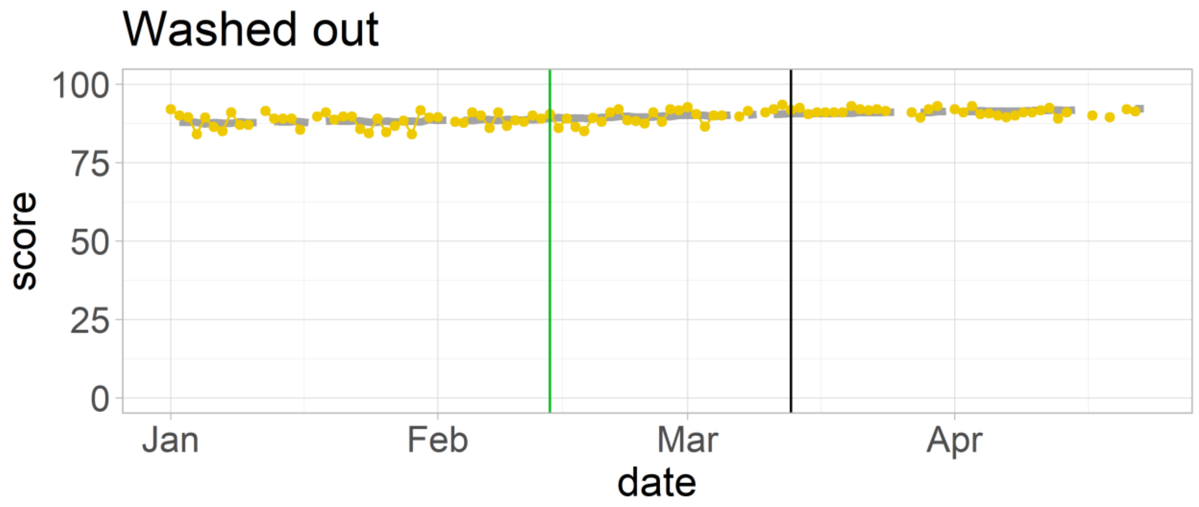
Washed out
100
75
50
25
0
score
Jan
Feb
Mar
Apr
date
